# Real-world safety of first-line enfortumab vedotin plus pembrolizumab in advanced urothelial carcinoma: evidence from VigiBase and FAERS

**DOI:** 10.3389/fimmu.2026.1759135

**Published:** 2026-06-18

**Authors:** Zuan Li, Han Qu, Shuowen Wang, Zizhen Liu, Zhenghua Wu, Shanshan Hu, Runjuan Yang, Qiying Yan, Dongying Wu, Yuefen Lou, Guorong Fan

**Affiliations:** 1 Department of Clinical Pharmacy, Shanghai General Hospital, Shanghai Jiao Tong University School of Medicine, Shanghai, China; 2 Department of Pharmacy, Shanghai Fourth People’s Hospital, School of Medicine, Tongji University, Shanghai, China; 3 School of Pharmacy, Shanghai Jiao Tong University, Shanghai, China; 4 School of Medicine, Shanghai University, Shanghai, China

**Keywords:** adverse drug events, disproportionality analysis, enfortumab vedotin, FAERS, pembrolizumab, time to onset, VigiBase

## Abstract

**Background:**

Compared with platinum-based chemotherapy, the combination of enfortumab vedotin and pembrolizumab (EV + Pembro) achieves higher durable response rates and improves survival outcomes in patients with locally advanced or metastatic urothelial carcinoma. As the first regimen pairing an immune checkpoint inhibitor with an antibody-drug conjugate, it offers a novel, effective option. Yet evidence has largely been derived from clinical trials, and robust, comprehensive real-world characterization of adverse drug events (ADEs) remains limited. It is therefore essential to examine recent real-world data to delineate the safety profile of EV + Pembro with greater precision.

**Methods:**

We conducted disproportionality analyses of ADE reports related to EV + Pembro from the FDA Adverse Event Reporting System (FAERS) and the WHO VigiBase. Signal detection algorithms, including Reporting Odds Ratio (ROR), Proportional Reporting Ratio (PRR), Multi-item Gamma Poisson Shrinker (MGPS), and Bayesian Confidence Propagation Neural Network (BCPNN), were used to identify significant safety signals. Additionally, the time to onset of ADEs was assessed.

**Results:**

A total of 892 reports from VigiBase and 1,698 reports from FAERS were analyzed across 25 system organ classes. Several previously unlisted ADEs in the product labeling were identified, such as dehydration, cholestasis, ileus, and tumor lysis syndrome. Subgroup analysis revealed that male patients were more prone to dehydration and renal impairment, while female patients were more likely to experience skin discoloration and alopecia. The median onset time for ADEs was 18 days, with males experiencing ADEs significantly earlier than females. Immune system disorders had the shortest median onset time, while surgical and medical procedures had the longest.

**Conclusion:**

This real-world analysis confirms known adverse reactions and identifies new safety signals for EV + Pembro. It also highlights differential risk profiles across subgroups, providing valuable insights for clinical practice. Further research is needed to validate these findings and refine patient management strategies.

## 1 Introduction

Bladder cancer ranks as the ninth most commonly diagnosed cancer worldwide, with approximately 614,000 new cases and 221,000 deaths reported globally in 2022, imposing a substantial global disease burden (1). Urothelial carcinoma, which accounts for approximately 90% of bladder cancer cases, is the most prevalent type and can occur in a broad range, from the renal pelvis to the proximal urethra (2). Advanced urothelial carcinoma (aUC) generally has a poor prognosis. Platinum-based chemotherapy has long been the standard first-line treatment for locally advanced or metastatic urothelial carcinoma (la/mUC). However, safety concerns and low long-term remission rates have presented significant limitations. About half of patients are ineligible for chemotherapy and have a poor prognosis, with a low five-year survival rate (3, 4). In recent years, the treatment landscape for la/mUC patients has rapidly evolved.

Enfortumab Vedotin (EV), an antibody-drug conjugate (ADC) targeting nectin-4, binds to cells expressing nectin-4 and releases monomethyl auristatin E (MMAE) through proteolytic cleavage. MMAE disrupts microtubules, inducing cell cycle arrest and apoptosis (5, 6). Pembrolizumab (Pembro) is an immune checkpoint inhibitor (ICI) that is a monoclonal antibody targeting the PD-1 receptor, thereby blocking its interaction with PD-L1 and PD-L2 and restoring antitumor T-cell activity. This blockade alleviates the inhibition of immune responses through the PD-1 pathway, reactivating the immune system and enhancing antitumor immunity (7). Building on early activity from the phase Ib/II EV-103 study, the U.S. FDA granted accelerated approval in 2023 to the combination of EV plus Pembro (EV + Pembro) for first-line treatment of la/mUC; subsequently, regular approval was supported by the phase III EV-302 (KEYNOTE-A39) trial, which demonstrated clinically meaningful efficacy with a manageable safety profile (8–10). Although EV + Pembro has shown meaningful therapeutic benefit in clinical practice, its safety profile remains insufficiently characterized, as most studies emphasize clinical trial data with limited real-world evaluation. To date, real-world safety evidence for EV + Pembro remains sparse, largely confined to small retrospective series and meeting abstracts, and there are no published pharmacovigilance studies using signal detection for this combination at a large scale (11). As a regimen that combines an ADC with an ICI, EV + Pembro presents a complex safety profile that includes immune-mediated adverse events (irAEs), organ-specific toxicities, and overlapping and potentially additive toxicities between the two agents.

To bridge this gap, this study makes use of global adverse event reporting databases, including the U.S. FDA Adverse Event Reporting System (FAERS) and the WHO VigiBase database, to conduct a systematic pharmacovigilance analysis. The aim is to systematically characterize the safety profile of EV + Pembro by analyzing adverse drug events (ADEs), uncovering new safety signals, and providing real-world evidence to support patient management strategies.

## 2 Methods

### 2.1 Data collection

VigiBase serves as the World Health Organization’s international repository for ADE reports related to medicinal products and vaccines, and is administered by the Uppsala Monitoring Centre (UMC). UMC collects and integrates anonymized drug safety reports from over 180 national pharmacovigilance centers participating in the WHO International Drug Monitoring Program (https://www.who-umc.org/vigibase/vigibase/). This extensive coverage enables the identification of rare and region-specific safety signals, making it an invaluable resource for pharmacovigilance studies. FAERS is a publicly accessible database established by the U.S. FDA, designed to collect and analyze ADEs associated with drugs and biologics. It contains drug safety reports submitted by patients, healthcare professionals, and pharmaceutical companies. We conducted a pharmacovigilance study on ADEs associated with EV + Pembro data from both VigiBase and FAERS.

**Figure 1:**
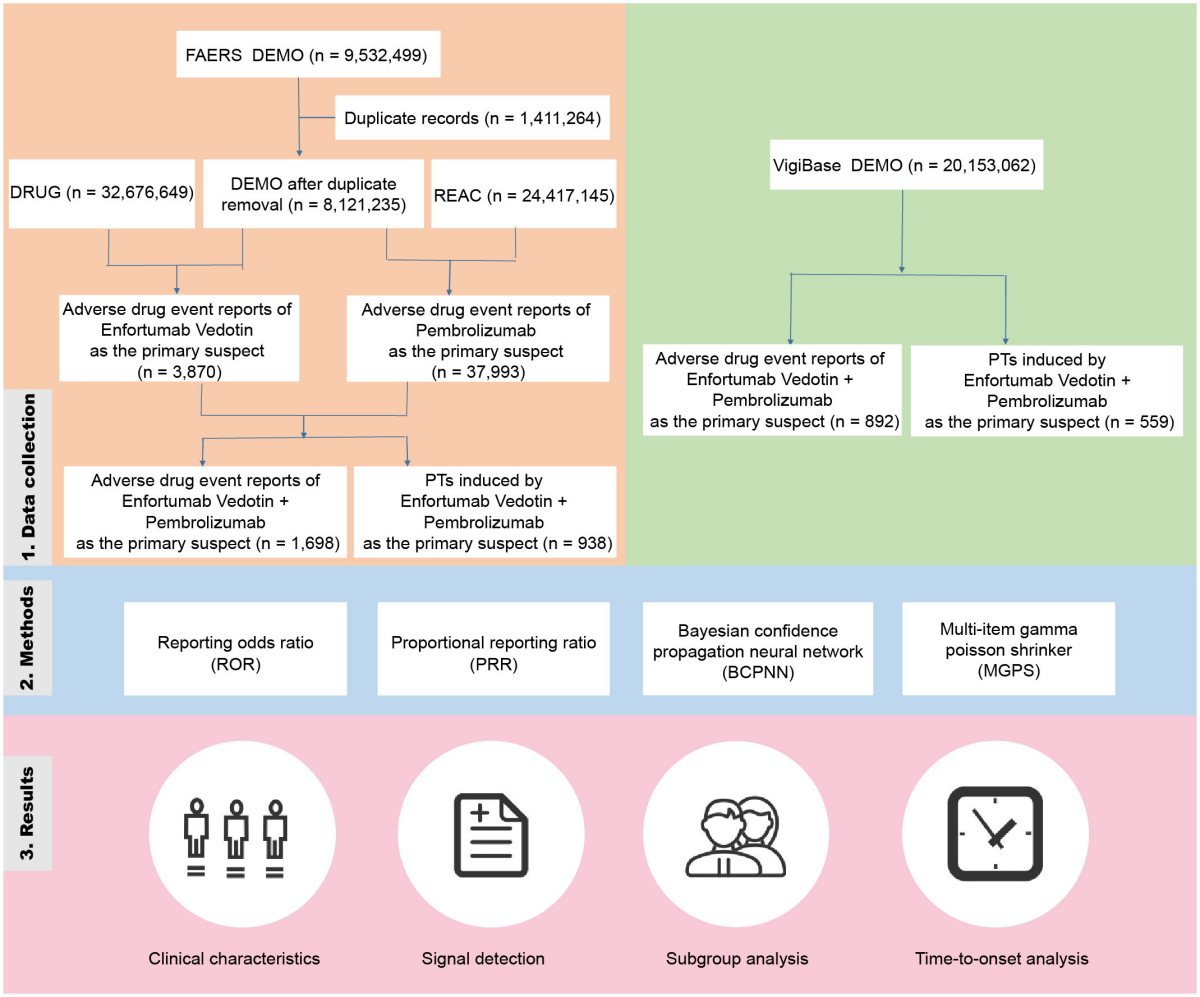
Workflow for processing EV + Pembro data in FAERS and VigiBase. The workflow shows data retrieval, deduplication, case selection, PT identification, and subsequent analyses, including clinical characteristics, signal detection, subgroup analysis, and time-to-onset analysis. DEMO, demographic and administrative information file; DRUG, drug information file; REAC, adverse reaction information file.

FAERS data were obtained free of charge from the U.S. FDA public website (12), while VigiBase data were accessed via VigiBase Custom Searches under a data use agreement with the UMC (13). We retrieved EV + Pembro-related data from VigiBase (from January 1, 2020, to July 1, 2025) and FAERS (from January 1, 2020, to June 30, 2025) for analysis. Reports involving EV + Pembro were retrieved using the generic and brand names of both drugs. The search terms for EV included “enfortumab vedotin” and “PADCEV,” and those for Pembro included “pembrolizumab” and “KEYTRUDA.” The Boolean search strategy was structured as follows: (“enfortumab vedotin” OR “PADCEV”) AND (“pembrolizumab” OR “KEYTRUDA”). To enhance the reliability of the results, the inclusion criterion was that EV was co-reported with Pembro, with at least one of the two drugs identified as the primary suspect. Duplicate reports in VigiBase were identified and removed using vigiMatch, an automated deduplication algorithm. FAERS reports were cleaned following the U.S. FDA’s recommended procedures. Specifically, duplicate reports were identified using CASEID, FDA_DT (FDA received date), and PRIMARYID. For reports with the same CASEID, only the record with the most recent FDA_DT was retained; when both CASEID and FDA_DT were identical, the report with the highest PRIMARYID was retained for analysis. The data processing flow is shown in **Figure 1**. ADEs were categorized using MedDRA Version 28.0 preferred terms (PTs) and grouped under corresponding system organ classes (SOCs). In this study, the disproportionality analysis evaluated all reported ADEs associated with EV + Pembro, regardless of severity. Among these reports, serious ADEs were further characterized to assess the clinical burden. According to FDA criteria, serious ADEs were defined as events leading to death, life-threatening conditions, initial or prolonged hospitalization, persistent or significant disability/incapacity, or other medically important events requiring intervention (14).

### 2.2 Disproportionality analysis

To identify drug safety signals, we performed disproportionality analyses using four established algorithms (15–18): Reporting Odds Ratio (ROR), Proportional Reporting Ratio (PRR), Bayesian Confidence Propagation Neural Network (BCPNN), and Multi-item Gamma Poisson Shrinker (MGPS). Among these, ROR is particularly effective at reducing bias for low-frequency events, while PRR offers higher specificity in signal detection. BCPNN can robustly identify signals, even with limited data, and MGPS is particularly adept at detecting rare ADE signals (19). These methods used a four-cell contingency table (**Supplementary Table S1**) to analyze the association between drug exposure and ADEs. The formulas for the proportional imbalance calculations are provided in **Supplementary Table S2**. Briefly, a signal was considered positive when it met the predefined threshold for the corresponding algorithm: lower limit of the 95% CI for ROR > 1 with N ≥ 3; PRR ≥ 2, χ^2^ ≥ 4, and N ≥ 3; IC025 > 0 for BCPNN; and EBGM05 > 2 for MGPS. IC025 was defined as the lower limit of the 95% credibility interval of the information component, and IC025 > 0 was considered indicative of a positive BCPNN signal. For rare or emerging label-unlisted ADEs, BCPNN positivity together with at least three reports was used as the screening criterion. We estimated subgroup‐specific disproportionality using ROR from a 2×2 contingency table. Two-sided P-values were obtained using the Pearson χ^2^ test under standard conditions; when any observed or expected cell count in the 2×2 table was <5, the Fisher’s exact test was used instead.

All data were processed and analyzed using Microsoft Excel 2021 (Microsoft Corporation, Redmond, WA, USA) and R software version 4.4.2 (R Foundation for Statistical Computing, Vienna, Austria). Visualizations were generated using R software version 4.4.2 and Python version 3.11.0 (Python Software Foundation, Wilmington, DE, USA).

### 2.3 Clinical seriousness classification

Descriptive clinical seriousness categories were assigned to label-unlisted ADEs to contextualize their potential clinical relevance. The classification was informed by FDA serious adverse event criteria and the general CTCAE severity framework (14, 20). Events were categorized as clinically serious, potentially serious, or usually non-serious but clinically relevant according to their usual clinical consequences and potential.

### 2.4 Time to onset analysis

In this analysis, TTO of ADEs related to EV + Pembro was defined as the interval between the ADE start date and the therapy start date. These dates were extracted from the FAERS DEMO and THER data files, respectively, which are provided as part of the FAERS quarterly data extracts. TTO values were standardized to days. TTO was summarized by the median and interquartile range (IQR) and modelled with the Weibull distribution. The flexibility of the Weibull distribution allows it to identify early (initial failure) and late (wear-out failure) risk patterns. The shape parameter (β) of the Weibull distribution provides insight into the risk trajectory over time: β < 1 indicates a decreasing risk, β = 1 a constant risk, and β > 1 an increasing risk. Log-rank tests were used to compare TTO distributions across ADE groups, and Kaplan–Meier curves were generated to visualize the cumulative incidence of ADEs over time.

## 3 Results

### 3.1 Clinical baseline characteristics

Clinical baseline characteristics are summarized in **Table 1**. We identified 892 EV + Pembro reports in VigiBase and 1,698 in FAERS. In both databases, reports predominantly involved male patients (n_VigiBase_ = 659 [73.9%]; n_FAERS_ = 1,219 [71.8%]), with females representing roughly one quarter (n_VigiBase_ = 208 [23.3%]; n_FAERS_ = 412 [24.3%]). Among records with age, older adults comprised the majority, particularly ≥65 years (n_VigiBase_ = 425 [72.0%]; n_FAERS_ = 851 [80.1%]). Regarding report sources, healthcare professionals, including doctors (n_VigiBase_ = 507, n_FAERS_ = 974), pharmacists (n_VigiBase_ = 46, n_FAERS_ = 134), and other healthcare providers (n_VigiBase_ = 165, n_FAERS_ = 290), accounted for the majority of all ADE reports (VigiBase: 80.5%, FAERS: 82.3%). Geographically, the Americas contributed the most reports in the VigiBase database (n = 469), followed by Europe (n = 332); in the FAERS database, Asia contributed the most reports (n = 778), followed by the Americas (n = 652). Among the reported outcomes, the most common were prolonged hospitalization (n_VigiBase_ = 184, n_FAERS_ = 482) and death (n_VigiBase_ = 90, n_FAERS_ = 314). Serious outcomes were frequent in both databases (VigiBase: 72.8%; FAERS: 88.9%).

**Table 1:** Clinical characteristics of EV + Pembro–related reports in FAERS and VigiBase.

Characteristics	VigiBase	FAERS
Total events	892	1698
Sex		
Female	208	412
Male	659	1219
Unknown	25	67
Age		
0–44	11	7
45–64	154	204
65–74	218	406
≥75	207	445
Unknown	302	636
Source		
Consumer	163	296
Other healthcare providers	165	290
Doctors	507	974
Pharmacists	46	134
Unknown	11	4
Region		
Africa	9	4
Americas	469	652
Asia	66	778
Europe	332	258
Oceania	16	6
Outcome		
Death	90	314
Disabling	5	9
Prolonged Hospitalization	184	482
Life-Threatening	14	45
Other Serious Outcomes	357	658
Serious Event Rate	72.8%	88.9%

### 3.2 Signal detection related to EV + pembro at the SOC level

**Figure 2:**
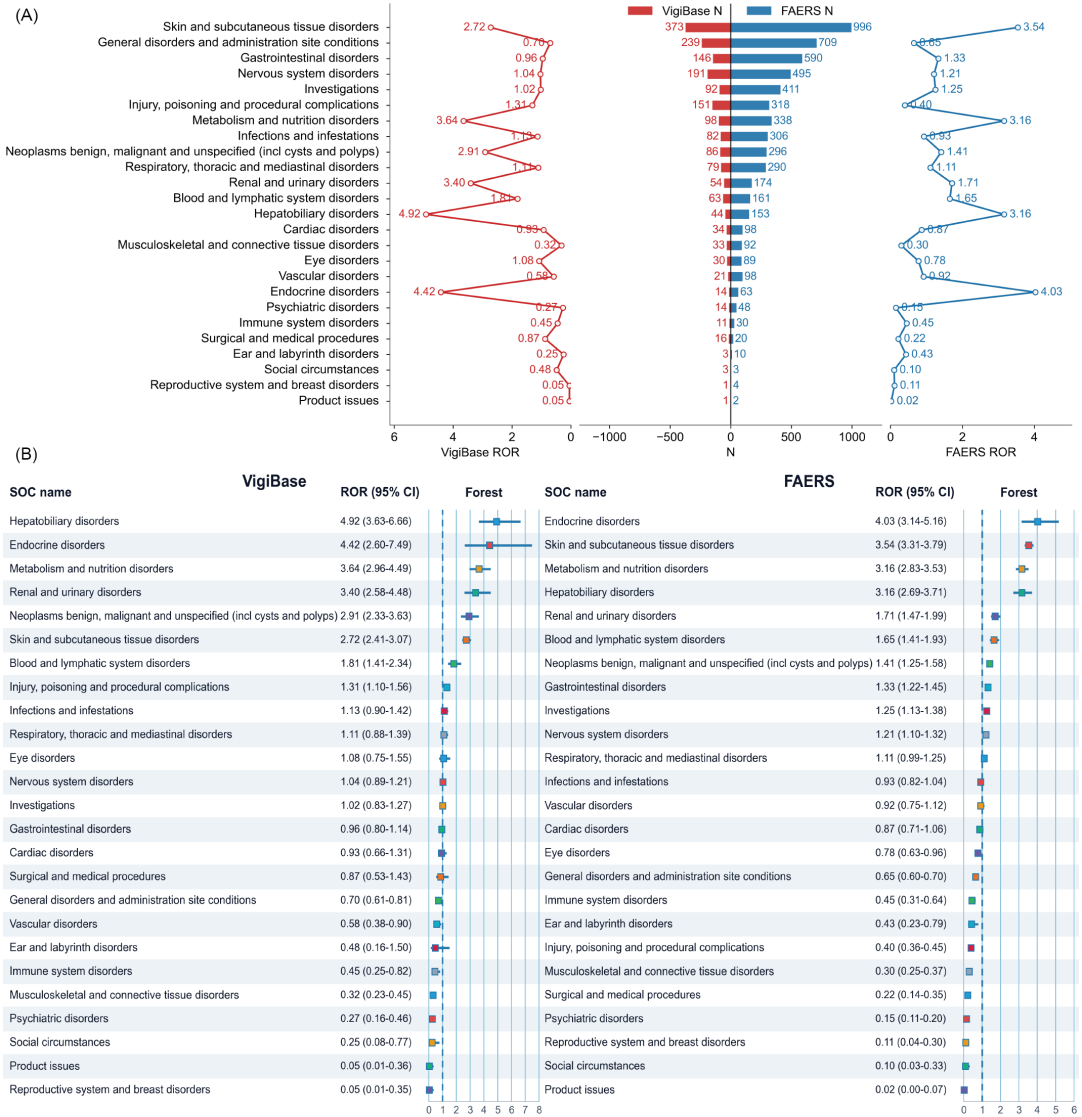
SOC-level ADE signals associated with EV + Pembro. **(A)** Distribution of ADE reports across 25 SOCs in VigiBase and FAERS, ranked by mean reporting frequency, with corresponding database-specific ROR values. **(B)** Forest plots showing database-specific RORs with 95% CIs for SOC-level ADE signals.

Our statistical analyses linked these signals to 25 SOCs and ranked them by the mean reporting frequency of ADEs and the ROR [95% CI] (**Figures 2A, B**). Overall, VigiBase and FAERS showed broadly consistent SOC patterns with overlapping categories at high risk, supporting the robustness of the findings; detailed SOC level data are provided in **Supplementary Table S3**.

In VigiBase, the most frequently reported SOC was skin and subcutaneous tissue disorders (n = 373); the top five SOCs by ROR were hepatobiliary disorders (ROR = 4.92 [3.63–6.66]), endocrine disorders (ROR = 4.42 [2.60–7.49]), metabolism and nutrition disorders (ROR = 3.64 [2.96–4.49]), renal and urinary disorders (ROR = 3.4 [2.58–4.48]), neoplasms benign, malignant and unspecified, including cysts and polyps (ROR = 2.91 [2.33–3.63]). Similarly, in the FAERS database, the most frequently reported SOC was skin and subcutaneous tissue disorders (n = 996); the five leading SOCs by ROR were endocrine disorders (ROR = 4.03 [3.14–5.16]), skin and subcutaneous tissue disorders (ROR = 3.54 [3.31–3.79]), hepatobiliary disorders (ROR = 3.16 [2.69–3.71]), metabolism and nutrition disorders (ROR = 3.16 [2.83–3.53]), and renal and urinary disorders (ROR = 1.71 [1.47–1.99]). Taken together, three SOCs, including endocrine disorders, hepatobiliary disorders, and metabolism and nutrition disorders, consistently exhibited elevated disproportionality across both databases; skin and subcutaneous tissue disorders were not only among the most frequently reported but also showed a strong signal; renal and urinary disorders were elevated in both datasets. Overall, these patterns delineate a coherent signal profile across databases and underscore the need for heightened clinical surveillance of these organ systems.

### 3.3 Signal detection related to EV + pembro at the PT level

**Figure 3:**
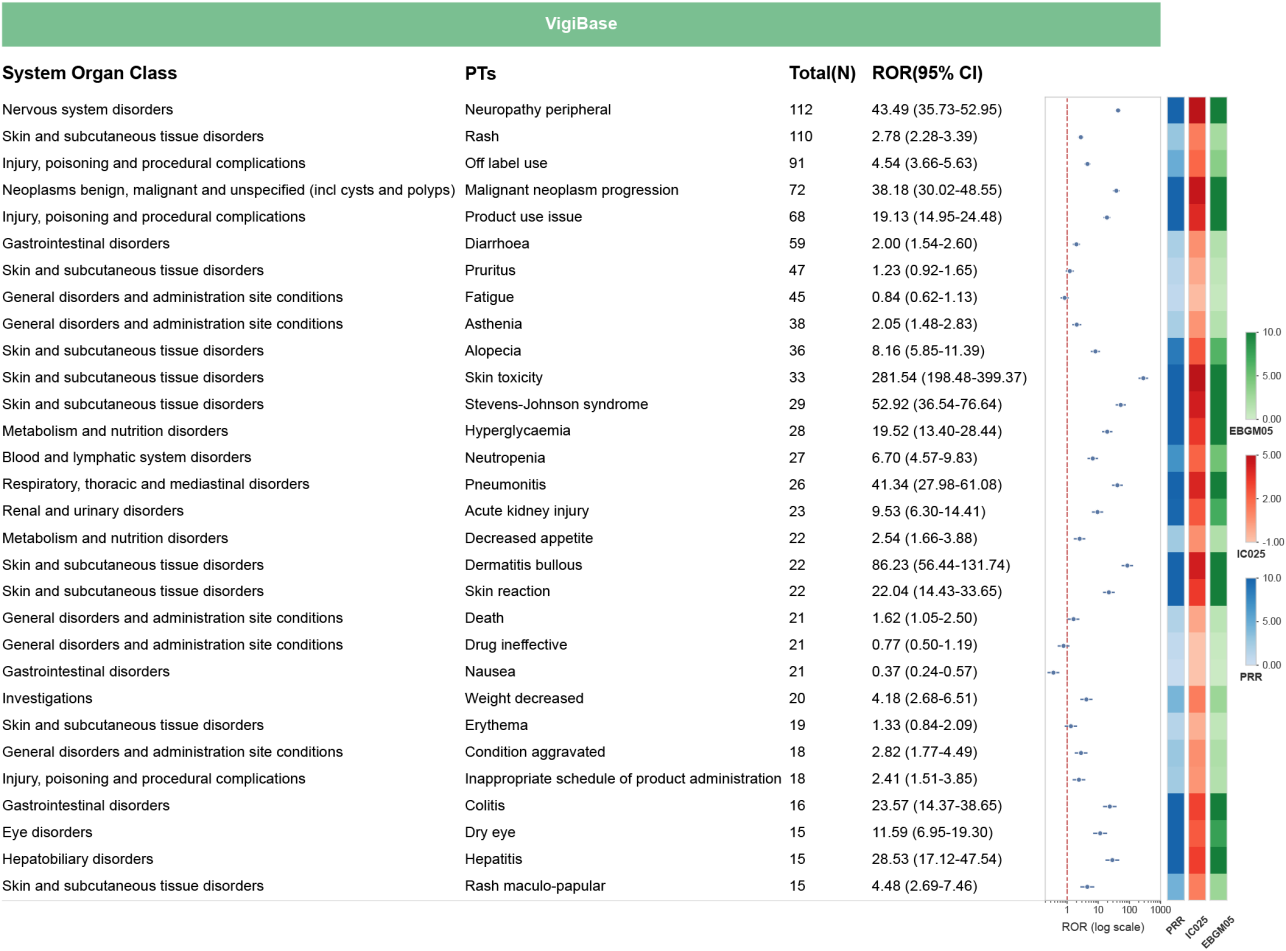
Top 30 PT-level ADEs ranked by reporting frequency in VigiBase. The table and forest plot show the RORs with 95% CIs for the top 30 PT-level ADEs in VigiBase, with adjacent heatmaps summarizing corresponding PRR, IC025, and EBGM05 signal metrics.

**Figure 4:**
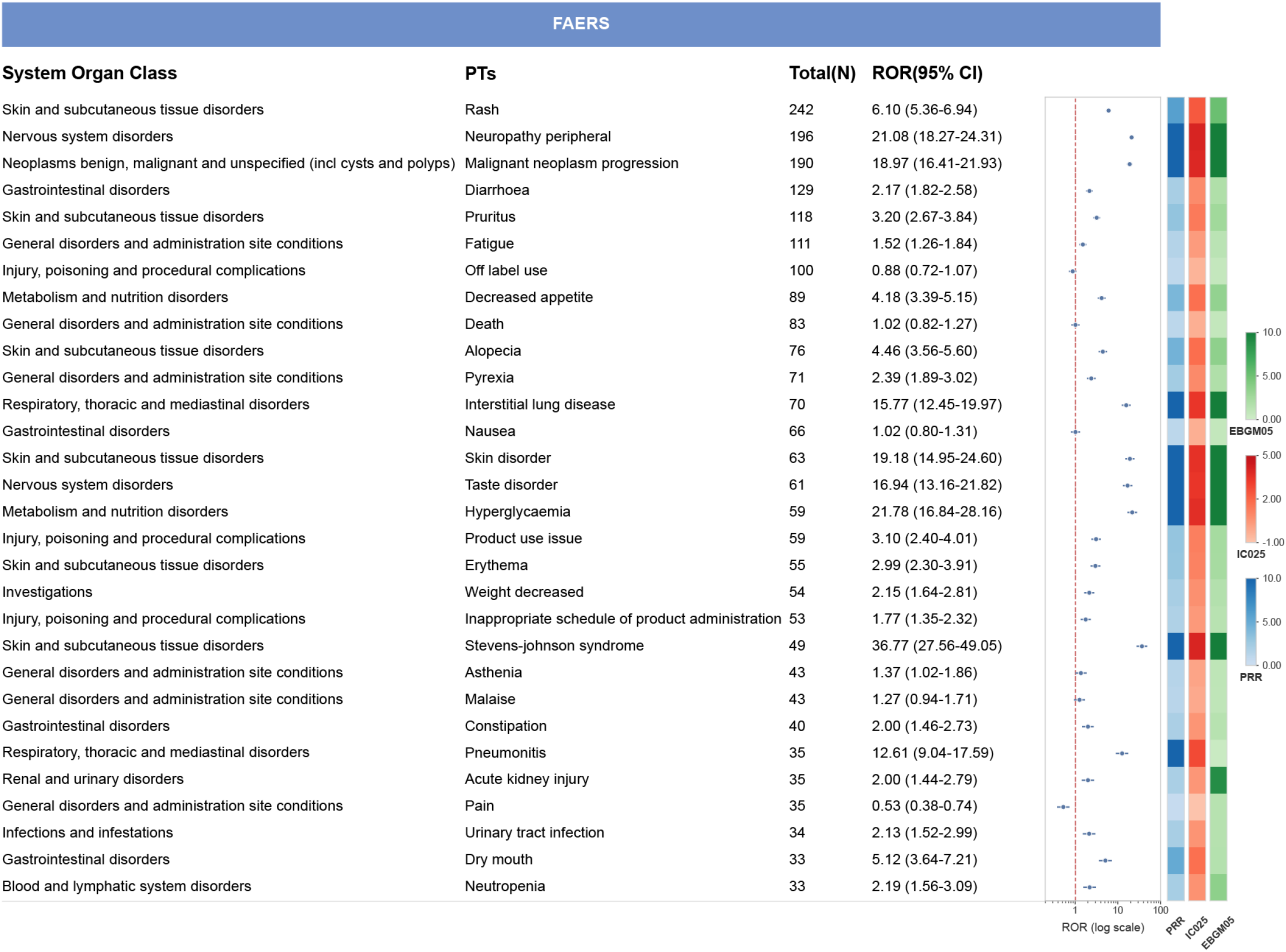
Top 30 PT-level ADEs ranked by reporting frequency in FAERS. The table and forest plot show the RORs with 95% CIs for the top 30 PT-level ADEs in FAERS, with adjacent heatmaps summarizing corresponding PRR, IC025, and EBGM05 signal metrics.

Our analyses identified 559 and 938 PTs in VigiBase and FAERS, respectively. **Figures 3** and **4** present forest plots of the top 30 PT-level ADEs ranked by reporting frequency in VigiBase and FAERS, respectively. Across both databases, a shared core of high-frequency events emerged: rash, peripheral neuropathy, diarrhea, pruritus, fatigue, and alopecia, with differences primarily in rank order. After excluding PTs considered unrelated to the drug or plausibly attributable to disease progression, the top ten PTs by reporting frequency in VigiBase were peripheral neuropathy (n = 112), rash (n = 110), diarrhea (n = 59), pruritus (n = 47), fatigue (n = 45), asthenia (n = 38), alopecia (n = 36), skin toxicity (n = 33), Stevens-Johnson syndrome (n = 29), and hyperglycemia (n = 28). In FAERS, the top ten were rash (n = 242), peripheral neuropathy (n = 196), diarrhea (n = 129), pruritus (n = 118), fatigue (n = 111), decreased appetite (n = 89), alopecia (n = 76), pyrexia (n = 71), interstitial lung disease (n = 70), and nausea (n = 66). These patterns are concordant with the product labeling, strengthening validity across databases and clinical plausibility.

Signal detection incorporated four complementary disproportionality methods (ROR, PRR, MGPS, and BCPNN). To enhance robustness and minimize false positives, we retained only PTs that met prespecified positivity thresholds in all four algorithms (intersection rule), yielding 101 PTs in VigiBase and 121 PTs in FAERS. We then prioritized the 30 highest ROR PTs for presentation in **Supplementary Tables S4**, **S5**. After filtering out PTs not attributable to treatment or indicative of disease progression, the ten highest ROR PTs in VigiBase were toxic erythema of chemotherapy (ROR = 461.08 [146.73–1448.90]), epidermal necrosis (ROR = 351.64 [130.69–946.14]), skin toxicity (ROR = 291.92 [205.8–414.1]), symmetrical drug-related intertriginous and flexural exanthema (ROR = 186.1 [76.99–449.84]), Stevens–Johnson syndrome/toxic epidermal necrolysis (SJS/TEN) overlap (ROR = 173.79 [64.84–465.83]), immune-mediated adverse reaction (ROR = 124.14 [39.84–386.84]), peripheral motor neuropathy (ROR = 122.79 [39.4–382.62]), insulin resistance (ROR = 103.64 [42.94–250.11]), dermatitis bullous (ROR = 88.33 [57.82–134.95]), and peripheral sensory neuropathy (ROR = 64.34 [32.05–129.19]). In FAERS, the ten leading PTs ranked by ROR were pleurisy bacterial (ROR = 632.2 [187.81–2128.11]), vascular access complication (ROR = 90.47 [44.89–182.34]), septic pulmonary embolism (ROR = 85.43 [27.23–268.01]), KL-6 increased (ROR = 78.05 [24.9–244.61]), immune-mediated nephritis (ROR = 69.12 [28.55–167.31]), Eastern Cooperative Oncology Group performance status worsened (ROR = 54.94 [29.43–102.58]), SJS/TEN overlap (ROR = 51.95 [23.22–116.26]), epidermal necrosis (ROR = 46.58 [17.38–124.82]), immune-mediated enterocolitis (ROR = 40.8 [27.49–60.55]), and dermatitis exfoliative (ROR = 38.75 [16.06–93.51]). Taken together, VigiBase and FAERS provided distinct but complementary high-ROR signals for EV + Pembro, with some overlap in immune-related and dermatologic toxicities. Severe cutaneous irAEs, notably SJS and TEN, showed strong and consistent disproportionality across both databases. While elevated RORs indicate disproportionate reporting rather than incidence or causality, these PTs warrant heightened clinical vigilance in patients receiving EV + Pembro. When reports are limited, the information component typically shows better ranking stability, supporting conservative signal identification. Our analysis found that ranking by information component values was largely consistent with the ordering based on ROR (**Supplementary Tables S6**, **S7**), reinforcing the robustness of the signals across independent algorithms and their relevance to clinical monitoring.

Using BCPNN positivity and at least three reports to probe rare or emerging events, we identified clinically relevant ADEs not currently listed in the product labeling across both databases, including dehydration, cholestasis, ascites, troponin increased, acute myocardial infarction, and skin discoloration. In FAERS, additional label-unlisted ADEs were observed with notable reporting frequencies (n > 9), including ileus, tumor lysis syndrome, and cerebral infarction. **Table 2** summarizes the report counts, proportions, IC (IC025) values, and descriptive clinical seriousness categories for these label-unlisted ADEs. These signals may help identify clinically relevant risks, guide ongoing safety monitoring, and inform future safety-labeling considerations pending further validation.

**Table 2:** Label-unlisted ADE signals and descriptive clinical seriousness classification.

PT name	VigiBase, n (%)	VigiBase, IC (IC025)	FAERS, n (%)	FAERS, IC (IC025)	Descriptive clinical seriousness category
Dehydration	9 (1.01%)	2.33 (0.67)	32 (1.88%)	1.72 (1.11)	Potentially serious
Cholestasis	3 (0.34%)	3.85 (2.18)	6 (0.35%)	1.81 (0.28)	Potentially serious
Ascites	3 (0.34%)	3.38 (1.71)	7 (0.41%)	1.59 (0.24)	Potentially serious
Troponin increased	3 (0.34%)	2.87 (1.20)	4 (0.24%)	2.42 (0.23)	Potentially serious
Acute myocardial infarction	3 (0.34%)	2.60 (0.93)	5 (0.29%)	1.53 (0.14)	Clinically serious
Skin discoloration	9 (1.01%)	2.74 (1.08)	11 (0.65%)	1.50 (0.46)	Usually non-serious but clinically relevant
Ileus	—	—	12 (0.71%)	3.79 (2.00)	Potentially serious
Tumor lysis syndrome	—	—	12 (0.71%)	3.68 (1.95)	Clinically serious
Cerebral infarction	—	—	9 (0.53%)	2.44 (1.00)	Clinically serious

Percentages represent the proportion of each ADE among all EV + Pembro reports in the corresponding database. IC (IC025) values are shown for BCPNN-based signal detection; IC025 > 0 indicates a positive signal. “—” indicates that no corresponding label-unlisted signal was identified in the database.

### 3.4 Subgroup analysis

**Figure 5:**
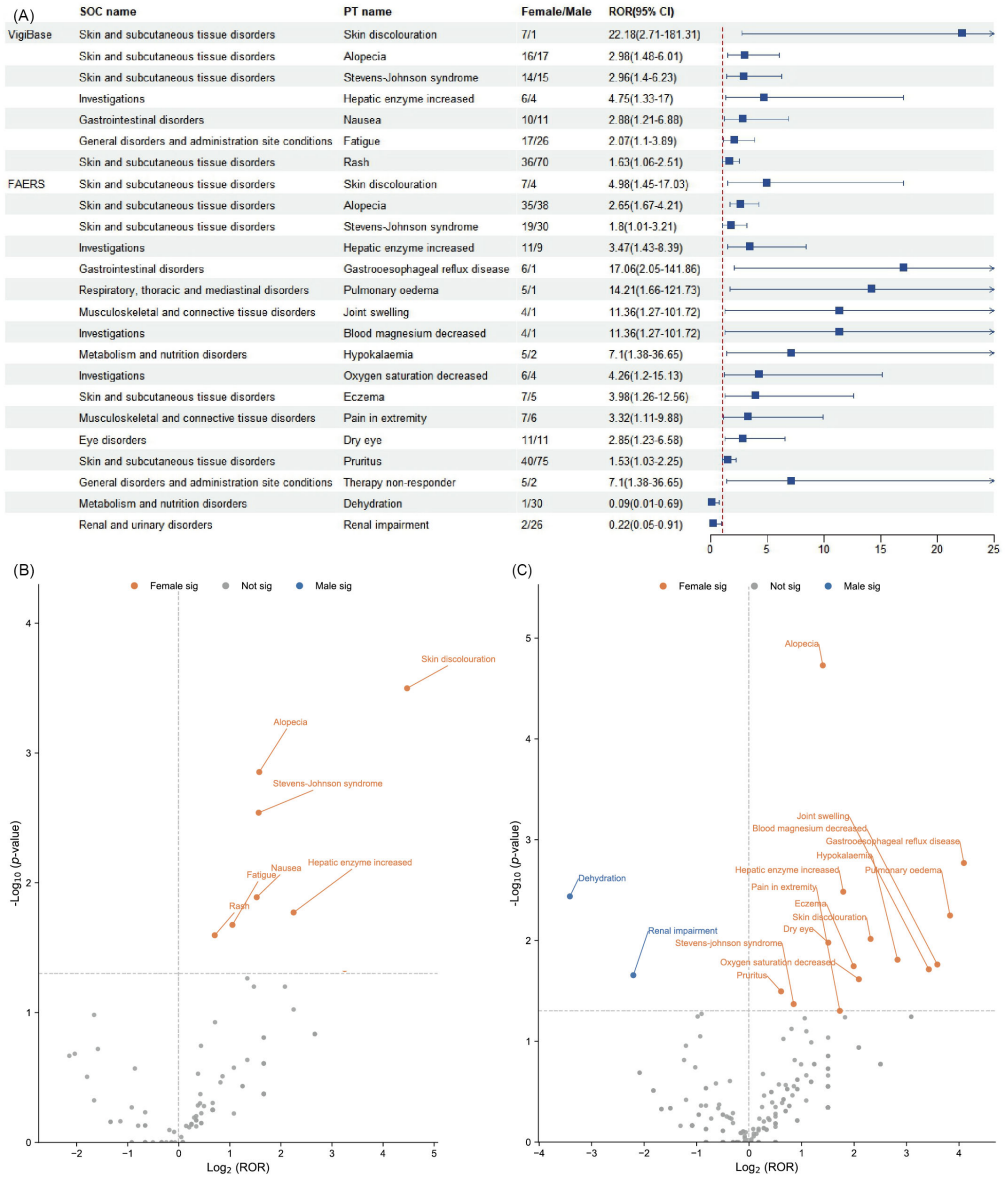
Sex-stratified ADE signals associated with EV + Pembro. **(A)** Female-to-male RORs with 95% CIs for sex-differentiated ADE signals. Volcano plots show the magnitude and statistical significance of sex-differentiated signals in VigiBase **(B)** and FAERS **(C)**. ROR > 1 indicates female-predominant reporting, whereas ROR < 1 indicates male-predominant reporting.

We examined sex differences in ADEs associated with EV + Pembro across both databases. Several PTs showed differences in reporting by sex; for example, increased blood creatinine was exclusively reported in males, whereas anemia of malignant disease was reported only in females (**Supplementary Table S8**). Disproportionality analyses estimated risks by sex using ROR (**Figure 5A**). To improve readability and avoid redundancy, representative sex-stratified ADE signals that were consistently observed across databases or considered clinically relevant are summarized in **Table 3**. Across both databases, alopecia, skin discoloration, SJS, and hepatic enzyme increased were more frequently reported in females. In addition, pulmonary oedema and joint swelling were also more common in females in FAERS, while rash was more common in females in VigiBase. In contrast, male-predominant signals were less frequent and were mainly observed in FAERS; dehydration and renal impairment were more frequently reported in males. Volcano plots highlight significant differences by sex in VigiBase (**Figure 5B**) and FAERS (**Figure 5C**). Detailed results are provided in **Supplementary Table S9**.

**Table 3:** Representative sex-differentiated ADE signals associated with EV + Pembro.

ADE	Database	Female/male, n	ROR (95% CI)	P value
Female-predominant signals				
Skin discoloration	VigiBase	7/1	22.18 (2.71–181.31)	0.00032
	FAERS	7/4	4.98 (1.45–17.03)	0.00965
Alopecia	VigiBase	16/17	2.98 (1.48–6.01)	0.00140
	FAERS	35/38	2.65 (1.67–4.21)	0.00002
Stevens–Johnson syndrome	VigiBase	14/15	2.96 (1.40–6.23)	0.00288
	FAERS	19/30	1.80 (1.01–3.21)	0.04272
Hepatic enzyme increased	VigiBase	6/4	4.75 (1.33–17.00)	0.01693
	FAERS	11/9	3.47 (1.43–8.39)	0.00328
Rash	VigiBase	36/70	1.63 (1.06–2.51)	0.02537
Joint swelling	FAERS	4/1	11.36 (1.27–101.72)	0.01828
Pulmonary oedema	FAERS	5/1	14.21 (1.66–121.73)	0.00565
Male-predominant signals				
Dehydration	FAERS	1/30	0.09 (0.01–0.69)	0.00365
Renal impairment	FAERS	2/26	0.22 (0.05–0.91)	0.02209

ADE, adverse drug event; CI, confidence interval; EV, enfortumab vedotin; FAERS, FDA Adverse Event Reporting System; Pembro, pembrolizumab; ROR, reporting odds ratio; SJS, Stevens–Johnson syndrome. RORs were calculated as female-to-male reporting odds ratios; ROR > 1 indicates female-predominant reporting, whereas ROR < 1 indicates male-predominant reporting. This table presents representative sex-stratified ADE signals; the complete results are provided in **Supplementary Table 9**.

### 3.5 TTO analysis

Understanding the TTO of ADEs is crucial for clinical decision-making. After excluding reports with missing, inaccurate, or erroneous start dates, we identified 570 EV + Pembro ADE reports with valid TTO in FAERS.

**Figure 6:**
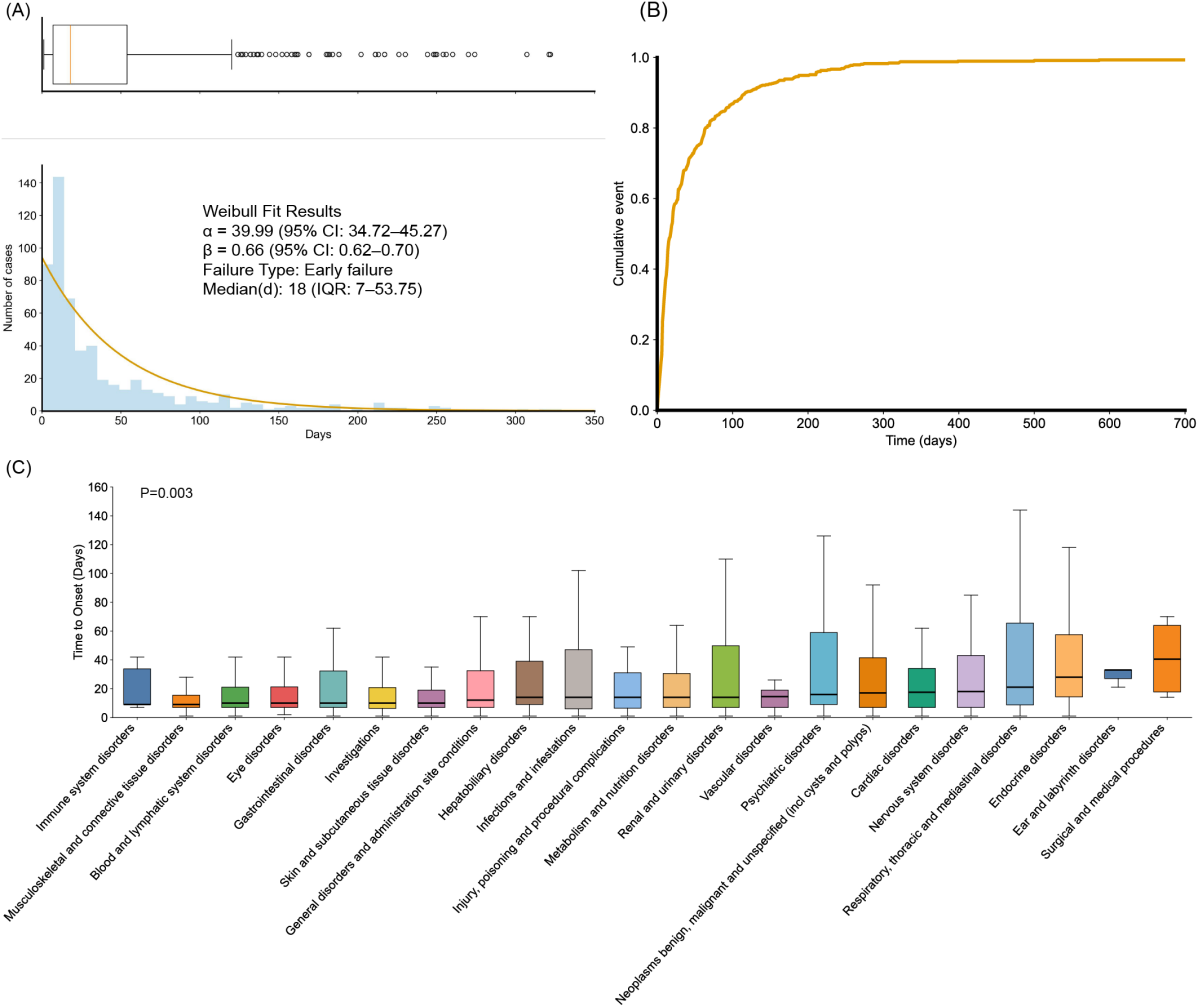
Time-to-onset distribution and SOC-level comparison of ADEs associated with EV + Pembro. **(A)** Overall TTO distribution shown by boxplot, histogram, and fitted Weibull curve. **(B)** Cumulative event curve of ADE onset over time. **(C)** SOC-level comparison of TTO, shown by boxplots.

**Figure 6A** displays the TTO histogram with a fitted Weibull distribution. The median TTO was 18 days (IQR, 7–53.75 days). Weibull analysis yielded a scale (α) of 39.99 (95% CI, 34.72–45.27) and a shape (β) of 0.66 (95% CI, 0.62–0.70); β < 1 indicates that the hazard decreases over time. This pattern is consistent with early failure risk, meaning ADEs are more likely to occur early in treatment. **Figure 6B** shows the cumulative incidence, which rises steeply early on and then plateaus. These results suggest that clinicians should prioritize ADE monitoring during the early phase of EV + Pembro therapy.

**Figure 7:**
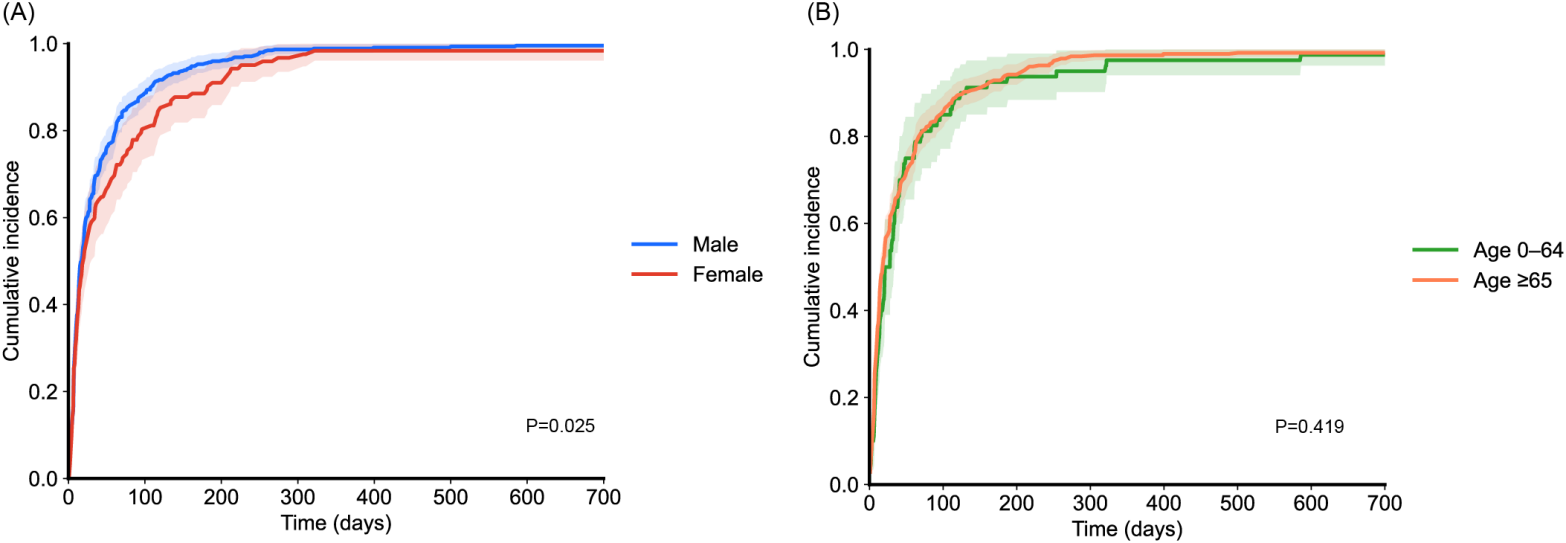
Time-to-onset analysis stratified by sex and age group. Kaplan–Meier cumulative event curves compare TTO distributions by sex **(A)** and age group **(B)**.

TTO differed significantly across SOCs (P = 0.003; see **Figure 6C**; **Supplementary Table S10**). Immune system disorders had the shortest median TTO (9 days), whereas surgical and medical procedures had the longest (40.5 days). We further stratified TTO by sex and age. Kaplan–Meier curves for males versus females (**Figure 7A**) indicated earlier onset in males (P = 0.025), while no significant difference by age group was observed (P = 0.419; **Figure 7B**).

## 4 Discussion

### 4.1 From trials to real-world: rationale for this study

EV + Pembro is now recommended as the preferred first-line therapy for untreated la/mUC. This position is endorsed by major guidelines, including those of the National Comprehensive Cancer Network (21), the European Society for Medical Oncology (3), and the European Association of Urology (22). EV + Pembro significantly prolongs overall survival and progression-free survival, marking a major advance in the management of la/mUC. Although neither agent is a traditional cytotoxic drug, both are associated with substantial toxicity. In a preference study of patients with genitourinary cancers, patients valued treatment experience, including ADEs related to therapy, over overall survival alone (23). Here, we integrate data from two complementary spontaneous reporting systems to characterize the real-world safety profile of EV + Pembro in advanced disease. The consistency between these two databases strengthens the robustness of the pharmacovigilance signals, while their complementary reporting characteristics provide a more complete real-world safety picture. Our goal was to quantify and describe known risks, identify and characterize drug safety signals, recognize populations at high risk, and promote rational clinical use of medications and risk prevention, providing evidence to inform public health policy and regulatory decision-making. To our knowledge, this is the first pharmacovigilance analysis to jointly evaluate ADEs related to EV + Pembro using both the FAERS and VigiBase databases.

### 4.2 Safety profile across databases

Baseline characteristics in VigiBase and FAERS were broadly consistent with the real-world la/mUC population. Most reports involved older male patients, consistent with epidemiology (22), and the predominance of reports from healthcare professionals (VigiBase: 80.5%; FAERS: 82.3%) suggests substantial clinician awareness of risks associated with EV + Pembro. Prolonged hospitalization (n_VigiBase_ = 184, n_FAERS_ = 482) and death (n_VigiBase_ = 90, n_FAERS_ = 314) were the most frequent reported outcomes. However, given the high disease burden and rapid progression of la/mUC, these outcomes may reflect underlying disease severity or disease progression rather than systemic treatment alone (24). Reporting patterns differed geographically, with VigiBase contributing more reports from Europe and the Americas and FAERS more from Asia, reflecting differences in reporting systems and reporting culture across regions.

### 4.3 SOC-level patterns and monitoring implications

The two databases showed high concordance in the distribution and signal patterns of SOCs related to the drug. Skin and subcutaneous tissue disorders were not only frequently reported but also displayed strong disproportionality in both databases (VigiBase: ROR = 2.72 [2.41–3.07]; FAERS: ROR = 3.54 [3.31–3.79]). Endocrine disorders (VigiBase: ROR = 4.42 [2.60–7.49]; FAERS: ROR = 4.03 [3.14–5.16]), hepatobiliary disorders (VigiBase: ROR = 4.92 [3.63–6.66]; FAERS: ROR = 3.16 [2.69–3.71]), metabolism and nutrition disorders (VigiBase: ROR = 3.64 [2.96–4.49]; FAERS: ROR = 3.16 [2.83–3.53]), and renal and urinary disorders (VigiBase: ROR = 3.40 [2.58–4.48]; FAERS: ROR = 1.71 [1.47–1.99]) likewise showed elevated disproportionality in both databases. This pattern may reflect additive toxicity mechanisms of EV + Pembro and indicates that these organ systems should be prioritized for clinical monitoring. EV carries an inherent risk of cutaneous toxicity, and Pembro is frequently accompanied by irAEs related to skin; when used together, they further elevate dermatologic risk. In EV-103 and EV-302, 70% of the 564 patients treated with EV + Pembro experienced skin reactions; the incidence, including severe events, was higher with the combination than with EV monotherapy (6). Although most reactions were low to moderate, a minority of severe events led to hospitalization and treatment interruption, underscoring the need for early standardized skin assessment and triage. Hepatobiliary disorders (VigiBase: ROR = 4.92 [3.63–6.66]; FAERS: ROR = 3.16 [2.69–3.71]), endocrine disorders (VigiBase: ROR = 4.42 [2.60–7.49]; FAERS: ROR = 4.03 [3.14–5.16]), and renal and urinary disorders (VigiBase: ROR = 3.40 [2.58–4.48]; FAERS: ROR = 1.71 [1.47–1.99]) emerged as notable safety signals, supporting routine laboratory monitoring at baseline and during early cycles, including liver and kidney function, blood glucose, and other standard tests, to manage reversible toxicities without compromising dose intensity.

### 4.4 PT-level signals under the combination therapy

At the PT level, our study identified a large number of ADEs associated with EV + Pembro, including rash, peripheral neuropathy, diarrhea, pruritus, and fatigue, which align with the drug label, indicating the reliability and stability of our findings (6). Disproportionality analysis revealed strong signals for SJS and TEN, which are included in the U.S. FDA boxed warning for EV and are also listed as serious immune-mediated reactions associated with Pembro. Therefore, proactive prevention, close monitoring, and timely management of severe skin reactions are critical. Patients should be educated to report symptoms immediately. In cases of suspected SJS/TEN, both offending agents should be promptly withdrawn, and early involvement of a specialist is imperative to optimize patient outcomes (25). In our analysis, peripheral neuropathy was one of the most frequently reported ADEs. Consistently, clinical trial data reported in the current product labeling showed that peripheral neuropathy occurred in 67% of patients receiving EV + Pembro (6). This is consistent with microtubule disruption in peripheral nerves mediated by MMAE; greater efficacy with the combination may lengthen EV exposure and increase neuropathy risk. Early identification and dose adjustment are essential to mitigate cumulative toxicity and maintain treatment continuity (26).

### 4.5 Label-unlisted signals: evidence and plausibility

We also identified potential ADEs not listed in current drug labels, including dehydration, cholestasis, ascites, troponin increased, acute myocardial infarction, skin discoloration, ileus, tumor lysis syndrome, and cerebral infarction. Pembro-related gastrointestinal inflammation or immune-mediated colitis (27), together with EV-related hyperglycemia and osmotic diuresis, may contribute to dehydration and subsequent renal injury. Pembro can also trigger immune-mediated hepatitis with cholestatic liver injury; if hepatic decompensation develops, ascites may occur through increased portal venous pressure and reduced serum albumin (28). Skin discoloration may be related to EV-associated injury in nectin-4-expressing skin structures and immune-mediated cutaneous effects (29, 30). ICIs have been linked to atherosclerotic events (31), and EV-related hyperglycemia may further promote hemoconcentration, coagulation factor elevation, and endothelial dysfunction (32), providing a plausible basis for troponin elevation, myocardial infarction, and cerebral infarction. Ileus occurs due to factors that disrupt normal intestinal motility, and its mechanism is complex, potentially life-threatening in severe cases (33). Previous pharmacovigilance studies have linked EV to gastrointestinal toxicity, and MMAE may cause autonomic neuropathy and gastrointestinal motility disorders (34). Furthermore, ICIs also induce gastrointestinal toxicity by disrupting the mucosal barrier and may lead to immune-related colitis or inflammatory bowel obstruction (35). Tumor lysis syndrome (TLS) results from rapid tumor cell death and the release of intracellular contents, leading to metabolic disturbances and acute kidney injury(AKI); because early manifestations can be nonspecific, TLS may be underrecognized in routine clinical care (36). A recent case report described TLS after EV + Pembro in aUC, underscoring the need for vigilant monitoring and prompt management (37).

Overall, these label-unlisted ADEs should be regarded as hypothesis-generating pharmacovigilance signals rather than evidence of causality. Their clinical relevance should be further evaluated through case-level clinical assessment, prospective studies, and mechanistic investigations.

### 4.6 Sex-stratified signals and clinical decision making

Sex stratified subgroup analysis showed that females were more likely to experience skin discoloration, rash, SJS, increased hepatic enzymes, pulmonary edema, joint swelling, and alopecia, whereas males were more prone to renal impairment and dehydration. Studies have indicated that males are more susceptible to AKI than females, potentially due to androgen-related RAAS activation, oxidative stress, and metabolic vulnerability (38, 39). In addition, ICI-associated hyperglycemia may lead to osmotic diuresis and dehydration, which can further contribute to renal injury (40).

Female immune responses are typically stronger, consistent with the higher prevalence of autoimmune disease in females (41), potentially increasing the burden of autoimmune-related conditions. Previous studies suggest that females may be more susceptible to immunotherapy-related toxicities, with higher risks of grade ≥3 adverse events, particularly symptomatic and hematologic events, and higher rates of skin irAEs and SJS/TEN; studies of PD-1 therapy further suggest that endocrine dysfunction, arthralgia, and pneumonitis are more frequent in females (42–45). Evidence shows that hair loss related to cancer is profoundly distressing, and females often experience a greater psychological burden related to appearance (46). This may make females more likely to report alopecia than males.

Because FAERS and VigiBase are spontaneous reporting databases, these sex-stratified findings may be influenced by reporting bias and incomplete clinical information. Therefore, they should be interpreted as hypothesis-generating signals rather than confirmatory evidence of true sex-specific risk. Nevertheless, clinicians may consider individualized monitoring based on patient sex, clinical characteristics, and overall risk profile, with closer attention to hydration status, electrolytes, and renal function in males, and to skin toxicity and immune-related events in females during EV + Pembro treatment.

### 4.7 TTO patterns and the early risk window

TTO analysis indicates that laboratory and clinical monitoring should be intensified during the first two months after initiating EV + Pembro to enable early detection and management of ADEs. The earlier onset of ADEs observed in males compared with females should be interpreted cautiously. Although MMAE is partly metabolized by CYP3A4 and sex-related differences in CYP3A4 activity have been reported (47), no specific data currently demonstrate higher MMAE exposure in males receiving EV + Pembro. Differences in baseline comorbidities, renal function, disease burden, treatment history, and reporting patterns may also have contributed to the observed TTO difference.

Our study revealed significant differences in TTO across SOCs. Immune system disorders showed the shortest TTO, possibly because EV may augment Pembro-mediated T cell responses, thereby synergistically amplifying early immune activity and advancing the onset (48). Surgical and medical procedures had the latest TTO, likely reflecting complications related to operative or interventional procedures during treatment. Because postoperative recovery is prolonged, complications related to the procedure may require a latency period before becoming clinically apparent.

We advocate stratification based on TTO for individualized ADE monitoring. Priority should be given to early immune-related toxicity, using scheduled symptom assessments and laboratory tests for timely intervention. Our TTO analysis supports ADEs surveillance and management. Future studies should combine real-world data with prospective validation across diverse cohorts to confirm TTO patterns and optimize management.

## 5 Limitations

This study has several limitations. Firstly, the FAERS and VigiBase databases are based on spontaneous reporting systems, which are inherently prone to underreporting and reporting bias. Such limitations may result in either overestimation or underestimation of specific ADEs. Not all ADEs are captured, and reporting patterns may be influenced by the perceptions and behaviors of healthcare providers and patients, thereby impacting the reliability of the safety evaluation. Furthermore, both databases lack comprehensive patient exposure data, making it difficult to accurately determine the incidence rates of ADEs. In addition, information on disease severity and prior local or systemic treatments was limited or unavailable, and these factors may have acted as potential confounders when interpreting reported ADEs and clinical outcomes. Consequently, assessing the true frequency of these events within the exposed population remains a challenge. While disproportionality analyses are useful for identifying statistical associations between drug exposures and reported ADEs, the observational design of these data sources precludes definitive conclusions regarding causality. Nevertheless, the potential safety signals identified in this study point to possible associations between the combination of EV and Pembro and specific ADEs. This large-scale, data-driven pharmacovigilance analysis provides meaningful insights that can support clinical decision-making and ongoing safety monitoring. Further prospective clinical investigations are warranted to confirm these findings and elucidate causal relationships.

## 6 Conclusion

Our analysis not only confirmed known ADEs but also identified previously unreported safety signals. Sex-based subgroup analysis revealed potential differences in ADE profiles between male and female patients. Additionally, our TTO analysis for both overall and subgroup data will help in monitoring and managing ADEs more effectively. However, the limited sample size of reported cases underscores the ongoing need for pharmacovigilance efforts and preclinical studies. Prospective clinical trials are necessary to validate these findings and optimize treatment outcomes for diverse patient populations.

## Data Availability

Publicly available datasets were analyzed in this study. This data can be found here: Access to VigiBase can be requested by contacting CustomSearches@who-umc.org. FAERS data are publicly accessible and were obtained from the official FDA website. (https://www.fda.gov/drugs/drug-approvals-and-databases/fda-adverse-event-reporting-system-faers-database).

## References

[1] Bray F Laversanne M Sung H Ferlay J Siegel RL Soerjomataram I . Global cancer statistics 2022: Globocan estimates of incidence and mortality worldwide for 36 cancers in 185 countries. CA: A Cancer J For Clin. (2024) 74:229–63. doi: 10.3322/caac.21834 38572751

[2] Chalasani V Chin JL Izawa JI . Histologic variants of urothelial bladder cancer and nonurothelial histology in bladder cancer. Can Urol Assoc J. (2009) 3:S193–8. doi: 10.5489/cuaj.1195 20019984 PMC2792446

[3] Powles T Bellmunt J Comperat E De Santis M Huddart R Loriot Y . Esmo clinical practice guideline interim update on first-line therapy in advanced urothelial carcinoma. Ann Oncol. (2024) 35:485–90. doi: 10.1016/j.annonc.2024.03.001 38490358

[4] Santoni M Rizzo A Massari F . Unlocking the mechanisms underlying the activity of pembrolizumab plus enfortumab vedotin in patients with urothelial carcinoma. Expert Opin Invest Drugs. (2025) 34:259–65. doi: 10.1080/13543784.2025.2473695 40012129

[5] Nikanjam M Pérez-Granado J Gramling M Larvol B Kurzrock R . Nectin-4 expression patterns and therapeutics in oncology. Cancer Lett. (2025) 622. doi: 10.1016/j.canlet.2025.217681 40209851 PMC12132789

[6] Seagen, Astellas Pharma Us Inc . Padcev (Enfortumab Vedotin-Ejfv) for Injection (2023). Available online at: https://Astellas.Us/Docs/Padcev_Label.Pdf (Accessed June 15, 2026).

[7] Merck & Co . Keytruda (Pembrolizumab) Injection (2024). Available online at: https://Www.Merck.Com/Product/Usa/Pi_Circulars/K/Keytruda/Keytruda_Pi.Pdf (Accessed June 15, 2026).

[8] Hoimes CJ Flaig TW Milowsky MI Friedlander TW Bilen MA Gupta S . Enfortumab vedotin plus pembrolizumab in previously untreated advanced urothelial cancer. J Clin Oncol. (2023) 41:22–31. doi: 10.1200/jco.22.01643 36041086 PMC10476837

[9] Powles T Valderrama BP Gupta S Bedke J Kikuchi E Hoffman-Censits J . Enfortumab vedotin and pembrolizumab in untreated advanced urothelial cancer. N Engl J Med. (2024) 390:875–88. doi: 10.1056/NEJMoa2312117 38446675

[10] Brave MH Maguire WF Weinstock C Zhang H Gao X Li F . Fda approval summary: Enfortumab vedotin plus pembrolizumab for locally advanced or metastatic urothelial carcinoma. Clin Cancer Res. (2024) 30:4815–21. doi: 10.1158/1078-0432.Ccr-24-1393 39230571 PMC11530298

[11] Jain P Naqvi SAA Tripathi N Oberoi JK Humayun MA Zakharia Y . Real-world outcomes of enfortumab vedotin and pembrolizumab in advanced urothelial carcinoma: A multicenter retrospective analysis. Clin Genitourin Cancer. (2025) 23:102453. doi: 10.1016/j.clgc.2025.102453 41177680

[12] U.S. Food and Drug Administration . Fda Adverse Event Reporting System (Faers) Database. Available online at: https://Www.Fda.Gov/Drugs/Drug-Approvals-and-Databases/Fda-Adverse-Event-Reporting-System-Faers-Database (Accessed June 15, 2026).

[13] Uppsala Monitoring Centre . Vigibase Search Services. Available online at: https://Who-Umc.Org/Vigibase-Data-Access/Vigibase-Search-Services/ (Accessed June 15, 2026).

[14] U.S. Food and Drug Administration . What is a Serious Adverse Event? Available online at: https://Www.Fda.Gov/Safety/Reporting-Serious-Problems-Fda/What-Serious-Adverse-Event (Accessed June 15, 2026).

[15] van Puijenbroek EP Bate A Leufkens HGM Lindquist M Orre R Egberts ACG . A comparison of measures of disproportionality for signal detection in spontaneous reporting systems for adverse drug reactions. Pharmacoepidemiol Drug Saf. (2002) 11:3–10. doi: 10.1002/pds.668 11998548

[16] Liu C Wang X Zhou C Cao X . A real-world disproportionality analysis of cidofovir from the Fda adverse event reporting system (Faers) database. Expert Opin Drug Saf. (2025) 24:1–9. doi: 10.1080/14740338.2025.2490271 40193180

[17] Bate A Lindquist M Edwards IR Olsson S Orre R Lansner A . A bayesian neural network method for adverse drug reaction signal generation. Eur J Clin Pharmacol. (1998) 54:315–21. doi: 10.1007/s002280050466 9696956

[18] Szarfman A MaChado SG O'Neill RT . Use of screening algorithms and computer systems to efficiently signal higher-than-expected combinations of drugs and events in the Us Fda's spontaneous reports database. Drug Saf. (2002) 25:381–92. doi: 10.2165/00002018-200225060-00001 12071774

[19] Gu J Qu Y Shen Y Zhou Q Jiang Y Zhu H . Comprehensive analysis of adverse events associated with pimavanserin using the Faers database. J Affect Disord. (2024) 362:742–8. doi: 10.1016/j.jad.2024.07.103 39029673

[20] National Cancer Institute . Common Terminology Criteria for Adverse Events (Ctcae), Version 6.0. Available online at: https://Dctd.Cancer.Gov/Research/Ctep-Trials/for-Sites/Adverse-Events/Ctcae-V6.Pdf (Accessed June 15, 2026).

[21] Flaig TW Spiess PE Abern M Agarwal N Bangs R Buyyounouski MK . Bladder cancer, version 3.2024. J Natl Compr Cancer Network. (2024) 22:216–25. doi: 10.6004/jnccn.2024.0024 38754471

[22] Eau Guidelines on Mibc. Introduction - Uroweb [Internet]. Uroweb - Eur. Assoc. Urol. [Cited 2025 October 30]. Available online at: https://Uroweb.Org/Guidelines/Muscle-Invasive-and-Metastatic-Bladder-Cancer (Accessed June 15, 2026).

[23] Benjamin DJ Rezazadeh Kalebasty A . Patient preferences in the treatment of genitourinary cancers. Nat Rev Urol. (2023) 20:513–4. doi: 10.1038/s41585-023-00765-8 37012440

[24] Kearney M Kirker M Thompson A Gharibian N Furegato M Pacheco C . Use of inpatient systemic chemotherapy and/or radiotherapy and related predictive factors, healthcare resource utilization, and direct hospitalization costs for metastatic urothelial cancer: Findings from a real-world retrospective observational study derived from the national hospital discharge claims database in Italy. BMC Cancer. (2024) 24:1470. doi: 10.1186/s12885-024-13075-y 39609723 PMC11606204

[25] Brower B McCoy A Ahmad H Eitman C Bowman IA Rembisz J . Managing potential adverse events during treatment with enfortumab vedotin + pembrolizumab in patients with advanced urothelial cancer. Front Oncol. (2024) 14. doi: 10.3389/fonc.2024.1326715 38711854 PMC11071165

[26] Sternschuss M Rosenberg JE . Enfortumab vedotin and pembrolizumab: Redefining the standard of care for previously untreated advanced urothelial cancer. Future Oncol. (2025) 21:1333–48. doi: 10.1080/14796694.2025.2482363 40129250 PMC12051594

[27] Cheng Y Ling F Li J Chen Y Xu M Li S . An updated review of gastrointestinal toxicity induced by Pd-1 inhibitors: From mechanisms to management. Front Immunol. (2023) 14:1190850. doi: 10.3389/fimmu.2023.1190850 37404814 PMC10315615

[28] Hountondji L Ferreira De Matos C Lebossé F Quantin X Lesage C Palassin P . Clinical pattern of checkpoint inhibitor-induced liver injury in a multicentre cohort. JHEP Rep. (2023) 5:100719. doi: 10.1016/j.jhepr.2023.100719 37138674 PMC10149360

[29] Lacouture ME Patel AB Rosenberg JE O'Donnell PH . Management of dermatologic events associated with the Nectin-4-directed antibody-drug conjugate enfortumab vedotin. Oncologist. (2022) 27:e223–32. doi: 10.1093/oncolo/oyac001 35274723 PMC8914492

[30] Wang E Kraehenbuehl L Ketosugbo K Kern JA Lacouture ME Leung DYM . Immune-related cutaneous adverse events due to checkpoint inhibitors. Ann Allergy Asthma Immunol. (2021) 126:613–22. doi: 10.1016/j.anai.2021.02.009 33609771 PMC8165024

[31] Piras L Zuccanti M Russo P Riccio F Agresti A Lustri C . Association between immune checkpoint inhibitors and atherosclerotic cardiovascular disease risk: Another brick in the wall. Int J Mol Sci. (2024) 25. doi: 10.3390/ijms25052502 38473748 PMC10931678

[32] Kaur R Kaur M Singh J . Endothelial dysfunction and platelet hyperactivity in type 2 diabetes mellitus: Molecular insights and therapeutic strategies. Cardiovasc Diabetol. (2018) 17:121. doi: 10.1186/s12933-018-0763-3 30170601 PMC6117983

[33] Catena F De Simone B Coccolini F Di Saverio S Sartelli M Ansaloni L . Bowel obstruction: A narrative review for all physicians. World J Emerg Surg. (2019) 14:20. doi: 10.1186/s13017-019-0240-7 31168315 PMC6489175

[34] Shi Y Yao K Zhao J Yue Y Wu H . Gastrointestinal toxicity of antibody-drug conjugates: A pharmacovigilance study using the Faers database. BMC Pharmacol Toxicol. (2025) 26:50. doi: 10.1186/s40360-025-00877-4 40033454 PMC11874441

[35] Dougan M . Gastrointestinal mucosal toxicities from immune checkpoint inhibitors: Current understanding and future directions. Immunol Rev. (2023) 318:11–21. doi: 10.1111/imr.13239 37455375

[36] Howard SC Avagyan A Workeneh B Pui CH . Tumour lysis syndrome. Nat Rev Dis Primers. (2024) 10:58. doi: 10.1038/s41572-024-00542-w 39174582

[37] Takemori D Shigehisa R Shimasaki S Yamashita E Kurano Y Atagi K . Successful management of tumor lysis syndrome following enfortumab vedotin plus pembrolizumab therapy in metastatic urothelial carcinoma: A case report. IJU Case Rep. (2025) 8:470–4. doi: 10.1002/iju5.70069 40909321 PMC12408168

[38] Loutradis C Pickup L Law JP Dasgupta I Townend JN Cockwell P . Acute kidney injury is more common in men than women after accounting for socioeconomic status, ethnicity, alcohol intake and smoking history. Biol Sex Differ. (2021) 12:30. doi: 10.1186/s13293-021-00373-4 33832522 PMC8034098

[39] Zhou P Gao Y Kong Z Wang J Si S Han W . Immune checkpoint inhibitors and acute kidney injury. Front Immunol. (2024) 15:1353339. doi: 10.3389/fimmu.2024.1353339 38464524 PMC10920224

[40] Chan JSK Lee S Kong D Lakhani I Ng K Dee EC . Risk of diabetes mellitus among users of immune checkpoint inhibitors: A population-based cohort study. Cancer Med. (2023) 12:8144–53. doi: 10.1002/cam4.5616 36647331 PMC10134274

[41] Klein SL Flanagan KL . Sex differences in immune responses. Nat Rev Immunol. (2016) 16:626–38. doi: 10.1038/nri.2016.90 27546235

[42] Unger JM Vaidya R Albain KS LeBlanc M Minasian LM Gotay CC . Sex differences in risk of severe adverse events in patients receiving immunotherapy, targeted therapy, or chemotherapy in cancer clinical trials. J Clin Oncol. (2022) 40:1474–86. doi: 10.1200/jco.21.02377 35119908 PMC9061143

[43] Bui AN Bougrine A Buchbinder EI Giobbie-Hurder A LeBoeuf NR . Female sex is associated with higher rates of dermatologic adverse events among patients with melanoma receiving immune checkpoint inhibitor therapy: A retrospective cohort study. J Am Acad Dermatol. (2022) 87:403–6. doi: 10.1016/j.jaad.2021.06.885 34252467

[44] Wasuwanich P So JM Chakrala TS Chen J Motaparthi K . Epidemiology of Stevens-Johnson syndrome and toxic epidermal necrolysis in the United States and factors predictive of outcome. JAAD Int. (2023) 13:17–25. doi: 10.1016/j.jdin.2023.06.014 37575514 PMC10413346

[45] Duma N Abdel-Ghani A Yadav S Hoversten KP Reed CT Sitek AN . Sex differences in tolerability to anti-programmed cell death protein 1 therapy in patients with metastatic melanoma and non-small cell lung cancer: Are we all equal? Oncologist. (2019) 24:e1148–55. doi: 10.1634/theoncologist.2019-0094 31036771 PMC6853107

[46] Dua P Heiland MF Kracen AC Deshields TL . Cancer-related hair loss: A selective review of the alopecia research literature. Psychooncology. (2017) 26:438–43. doi: 10.1002/pon.4039 26594010

[47] Wolbold R Klein K Burk O Nüssler AK Neuhaus P Eichelbaum M . Sex is a major determinant of Cyp3a4 expression in human liver. Hepatology. (2003) 38:978–88. doi: 10.1053/jhep.2003.50393 14512885

[48] Taylor C Patterson KM Friedman D Bacot SM Feldman GM Wang T . Mechanistic insights into the successful development of combination therapy of enfortumab vedotin and pembrolizumab for the treatment of locally advanced or metastatic urothelial cancer. Cancers (Basel). (2024) 16. doi: 10.3390/cancers16173071 39272928 PMC11393896

