# Supplementary material for: Real-world safety of first-line enfortumab vedotin plus pembrolizumab in advanced urothelial carcinoma: evidence from VigiBase and FAERS
Source: Front Immunol. 2026 Jun 18;17:1759135. doi: 10.3389/fimmu.2026.1759135 (PMC13323251; doi:10.3389/fimmu.2026.1759135)
Supplement: Supplementary file 1 [file Table1.docx]

***Supplementary Material***

**Supplementary Table S1. Two-by-two (2×2) contingency table for disproportionality analysis**

**Supplementary Table S2. Four major algorithms used for signal detection**

**Supplementary Table S3. SOC-level summary of EV + Pembro signals in VigiBase and FAERS**

**Supplementary Table S4. Top 30 PT-level ADEs sorted by ROR for EV + Pembro in VigiBase**

**Supplementary Table S5. Top 30 PT-level ADEs sorted by ROR for EV + Pembro in FAERS**

**Supplementary Table S6. Top 30 PT-level ADEs sorted by IC for EV + Pembro in VigiBase**

**Supplementary Table S7. Top 30 PT-level ADEs sorted by IC for EV + Pembro in FAERS**

**Supplementary Table S8. Sex-exclusive PTs by database (male-only vs female-only)**

**Supplementary Table S9. Analysis of sex-differentiated risk signals in EV + Pembro**

**Supplementary Table S10. TTO by SOC in FAERS (median and IQR)**

**Supplementary Table S1. Two-by-two (2×2) contingency table for disproportionality analysis**

|  | Target ADEs | Other ADEs | Total |
| --- | --- | --- | --- |
| EV + Pembro | a | b | a + b |
| Non-[combination therapy](https://zhida.zhihu.com/search?content_id=186695474&content_type=Article&match_order=1&q=combination+therapy&zhida_source=entity) | c | d | c + d |
| Total | a + c | b + d | a+b+c+d |

Equation: a, number of reports containing both EV + Pembro and the target ADE; b, number of reports containing EV + Pembro and other ADEs; c, number of reports containing the target ADE with non-combination therapies; d, number of reports containing non-combination therapies and other ADEs. ADE, adverse drug event; EV, enfortumab vedotin; Pembro, pembrolizumab.

**Supplementary Table S2. Four major algorithms used for signal detection**

| Algorithms | Equation | Criteria |
| --- | --- | --- |
| ROR | ROR=ad/bc | lower limit of 95% CI>1, N≥3 |
|  | 95%CI=e^ln(ROR)±1.96(1/a+1/b+1/c+1/d)^0.5^ |  |
| PRR | PRR=a(c+d)/c(a+b) | PRR≥2, χ^2^≥4, N≥3 |
|  | χ^2^=[(ad-bc)^2](a+b+c+d)/[(a+b)(c+d)(a+c)(b+d)] |  |
| BCPNN | IC=log_2_[a(a+b+c+d)/((a+b)(a+c))] | IC025>0 |
|  | 95%CI= E(IC) ± 2V(IC)^0.5 |  |
| MGPS | EBGM=a(a+b+c+d)/[(a+b)(a+c)] | EBGM05>2 |
|  | 95%CI=e^ln(EBGM)±1.96(1/a+1/b+1/c+1/d)^0.5^ |  |

Abbreviations: 95% CI, 95% confidence interval; N, the number of reports; χ2, chi-squared; IC, information component; IC025, the lower limit of the 95% credibility interval of the information component; E(IC), the IC, expectations; V(IC), the variance of IC; EBGM, empirical Bayes geometric mean; EBGM05, the lower limit of 95% CI, of EBGM. ROR, reporting odds ratio; PRR, proportional reporting ratio; BCPNN, Bayesian confidence propagation neural network; MGPS, multi-item gamma Poisson shrinker.

**Supplementary Table S3. SOC-level summary of EV + Pembro signals in VigiBase and FAERS**

| **SOC** | **VigiBase N** | **VigiBase**  **ROR(95% CI)** | **FAERS N** | **FAERS  ROR(95% CI)** |
| --- | --- | --- | --- | --- |
| Skin and subcutaneous tissue disorders | 373 | 2.72 (2.41–3.07) | 996 | 3.54 (3.31–3.79) |
| General disorders and administration site conditions | 239 | 0.70 (0.61–0.81) | 709 | 0.65 (0.60–0.70) |
| Gastrointestinal disorders | 146 | 0.96 (0.80–1.14) | 590 | 1.33 (1.22–1.45) |
| Nervous system disorders | 191 | 1.04 (0.89–1.21) | 495 | 1.21 (1.10–1.32) |
| Investigations | 92 | 1.02 (0.83–1.27) | 411 | 1.25 (1.13–1.38) |
| Injury, poisoning and procedural complications | 151 | 1.31 (1.10–1.56) | 318 | 0.40 (0.36–0.45) |
| Metabolism and nutrition disorders | 98 | 3.64 (2.96–4.49) | 338 | 3.16 (2.83–3.53) |
| Infections and infestations | 82 | 1.13 (0.90–1.42) | 306 | 0.93 (0.82–1.04) |
| Neoplasms benign, malignant and unspecified (incl cysts and polyps) | 86 | 2.91 (2.33–3.63) | 296 | 1.41 (1.25–1.58) |
| Respiratory, thoracic and mediastinal disorders | 79 | 1.11 (0.88–1.39) | 290 | 1.11 (0.99–1.25) |
| Renal and urinary disorders | 54 | 3.40 (2.58–4.48) | 174 | 1.71 (1.47–1.99) |
| Blood and lymphatic system disorders | 63 | 1.81 (1.41–2.34) | 161 | 1.65 (1.41–1.93) |

| **SOC** | **VigiBase N** | **VigiBase**  **ROR(95% CI)** | **FAERS N** | **FAERS  ROR(95% CI)** |
| --- | --- | --- | --- | --- |
| Hepatobiliary disorders | 44 | 4.92 (3.63–6.66) | 153 | 3.16 (2.69–3.71) |
| Cardiac disorders | 34 | 0.93 (0.66–1.31) | 98 | 0.87 (0.71–1.06) |
| Musculoskeletal and connective tissue disorders | 33 | 0.32 (0.23–0.45) | 92 | 0.30 (0.25–0.37) |
| Eye disorders | 30 | 1.08 (0.75–1.55) | 89 | 0.78 (0.63–0.96) |
| Vascular disorders | 21 | 0.58 (0.38–0.90) | 98 | 0.92 (0.75–1.12) |
| Endocrine disorders | 14 | 4.42 (2.60–7.49) | 63 | 4.03 (3.14–5.16) |
| Psychiatric disorders | 14 | 0.27 (0.16–0.46) | 48 | 0.15 (0.11–0.20) |
| Immune system disorders | 11 | 0.45 (0.25–0.82) | 30 | 0.45 (0.31–0.64) |
| Surgical and medical procedures | 16 | 0.87 (0.53–1.43) | 20 | 0.22 (0.14–0.35) |
| Ear and labyrinth disorders | 3 | 0.48 (0.16–1.50) | 10 | 0.43 (0.23–0.79) |
| Social circumstances | 3 | 0.25 (0.08–0.77) | 3 | 0.10 (0.03–0.33) |
| Reproductive system and breast disorders | 1 | 0.05 (0.01–0.35) | 4 | 0.11 (0.04–0.30) |
| Product issues | 1 | 0.05 (0.01–0.36) | 2 | 0.02 (0.00–0.07) |

**Supplementary Table S4. Top 30 PT-level ADEs sorted by ROR for EV + Pembro in VigiBase**

| **SOC name** | **PT** | **N** | **ROR (95% CI)** | **PRR (****χ^2^)** | **EBGM (EBGM05)** | **IC (IC025)** |
| --- | --- | --- | --- | --- | --- | --- |
| Skin and subcutaneous tissue disorders | Toxic erythema of chemotherapy | 3 | 461.08  (146.73-1448.9) | 459.54 (1345.19) | 450.37 (172.78) | 8.81 (7.13) |
| Skin and subcutaneous tissue disorders | Epidermal necrosis | 4 | 351.64  (130.69-946.14) | 350.08 (1371.0) | 344.73 (150.59) | 8.43 (6.75) |
| Skin and subcutaneous tissue disorders | Skin toxicity | 33 | 291.92  (205.8-414.1) | 281.54 (9108.59) | 277.97 (207.47) | 8.12 (6.45) |
| Skin and subcutaneous tissue disorders | Symmetrical drug-related intertriginous and flexural exanthema | 5 | 186.1  (76.99-449.84) | 185.07 (907.94) | 183.57 (87.72) | 7.52 (5.85) |
| Skin and subcutaneous tissue disorders | SJS–TEN overlap | 4 | 173.79  (64.84-465.83) | 173.02 (678.91) | 171.71 (75.25) | 7.42 (5.75) |
| Immune system disorders | Immune-mediated adverse reaction | 3 | 124.14  (39.84-386.84) | 123.73 (363.21) | 123.05 (47.54) | 6.94 (5.27) |
| Nervous system disorders | Peripheral motor neuropathy | 3 | 122.79  (39.4-382.62) | 122.38 (359.22) | 121.72 (47.03) | 6.93 (5.25) |
| Metabolism and nutrition disorders | Insulin resistance | 5 | 103.64  (42.94-250.11) | 103.07 (503.1) | 102.6  (49.09) | 6.68 (5.01) |
| Skin and subcutaneous tissue disorders | Dermatitis bullous | 22 | 88.33  (57.82-134.95) | 86.23 (1846.63) | 85.9  (60.25) | 6.42 (4.76) |
| Surgical and medical procedures | Radiotherapy | 4 | 78.93  (29.51-211.12) | 78.58 (305.32) | 78.31  (34.38) | 6.29 (4.62) |
| Nervous system disorders | Peripheral sensory neuropathy | 8 | 64.34  (32.05-129.19) | 63.78 (493.05) | 63.6  (35.5) | 5.99 (4.32) |
| Hepatobiliary disorders | Immune-mediated hepatitis | 4 | 63.96  (23.92-171.02) | 63.68 (246.09) | 63.5  (27.88) | 5.99 (4.32) |
| Skin and subcutaneous tissue disorders | Toxic epidermal necrolysis | 15 | 62.26  (37.35-103.79) | 61.25 (886.79) | 61.08  (39.83) | 5.93 (4.26) |

**Supplementary Table S4. (Continued)**

| **SOC name** | **PT** | **N** | **ROR (95% CI)** | **PRR (χ^2^)** | **EBGM (EBGM05)** | **IC (IC025)** |
| --- | --- | --- | --- | --- | --- | --- |
| Respiratory, thoracic and mediastinal disorders | Immune-mediated lung disease | 4 | 56.1  (20.98-149.98) | 55.85 (214.96) | 55.72  (24.47) | 5.8  (4.13) |
| Skin and subcutaneous tissue disorders | Stevens-Johnson syndrome | 29 | 54.61  (37.71-79.08) | 52.92 (1474.58) | 52.8  (38.73) | 5.72 (4.05) |
| Nervous system disorders | Neuropathy peripheral | 112 | 48.83  (40.11-59.44) | 43.49 (4651.71) | 43.4 (36.82) | 5.44 (3.77) |
| General disorders and administration site conditions | Therapy partial responder | 12 | 45.42  (25.68-80.33) | 44.83 (513.36) | 44.74 (27.77) | 5.48 (3.81) |
| Skin and subcutaneous tissue disorders | Dermatitis exfoliative | 4 | 43.34  (16.22-115.85) | 43.16 (164.41) | 43.07 (18.92) | 5.43 (3.76) |
| Respiratory, thoracic and mediastinal disorders | Pneumonitis | 26 | 42.52  (28.78-62.82) | 41.34 (1022.29) | 41.27 (29.77) | 5.37 (3.7) |
| Nervous system disorders | Polyneuropathy | 13 | 42.22  (24.41-73.04) | 41.63 (514.74) | 41.56 (26.27) | 5.38 (3.71) |
| Neoplasms benign, malignant and unspecified (incl cysts and polyps) | Malignant neoplasm progression | 72 | 41.18  (32.38-52.37) | 38.18 (2607.02) | 38.11 (31.17) | 5.25 (3.58) |
| Respiratory, thoracic and mediastinal disorders | Organising pneumonia | 3 | 37.28  (11.99-115.93) | 37.16 (105.4) | 37.1 (14.36) | 5.21 (3.54) |
| Metabolism and nutrition disorders | Diabetic ketoacidosis | 12 | 34.11  (19.29-60.32) | 33.67 (380.0) | 33.62 (20.87) | 5.07 (3.4) |
| Infections and infestations | Urosepsis | 3 | 29.66  (9.54-92.22) | 29.57 (82.7) | 29.53 (11.43) | 4.88 (3.21) |
| General disorders and administration site conditions | Infusion site extravasation | 5 | 29.12  (12.08-70.18) | 28.97 (134.85) | 28.93 (13.86) | 4.85 (3.19) |
| Hepatobiliary disorders | Hepatitis | 15 | 28.99  (17.4-48.31) | 28.53 (398.19) | 28.49 (18.59) | 4.83 (3.16) |

**Supplementary Table S4. (Continued)**

| **SOC name** | **PT** | **N** | **ROR (95% CI)** | **PRR (χ^2^)** | **EBGM (EBGM05)** | **IC (IC025)** |
| --- | --- | --- | --- | --- | --- | --- |
| Respiratory, thoracic and mediastinal disorders | Lung opacity | 5 | 28.33  (11.75-68.26) | 28.17 (130.91) | 28.14  (13.48) | 4.81  (3.15) |
| Nervous system disorders | Myasthenia gravis | 5 | 27.8  (11.54-67.0) | 27.65 (128.32) | 27.62  (13.23) | 4.79  (3.12) |
| Metabolism and nutrition disorders | Hypophosphatemia | 4 | 27.33  (10.23-73.02) | 27.21 (100.88) | 27.18  (11.94) | 4.76  (3.09) |
| General disorders and administration site conditions | Multiple organ dysfunction syndrome | 9 | 24.56  (12.73-47.38) | 24.33 (201.19) | 24.3  (14.03) | 4.6  (2.93) |

**Supplementary Table S5. Top 30 PT-level ADEs sorted by ROR for EV + Pembro in FAERS**

| **SOC name** | **PT** | **N** | **ROR (95% CI)** | **PRR (χ^2^)** | **EBGM (EBGM05)** | **IC (IC025)** |
| --- | --- | --- | --- | --- | --- | --- |
| Infections and infestations | Pleurisy bacterial | 3 | 632.2  (187.81-2128.11) | 631.87 (1643.15) | 549.58 (163.27) | 9.1  (0.43) |
| Neoplasms benign, malignant and unspecified (incl cysts and polyps) | Transitional cell carcinoma metastatic | 5 | 585.57  (229.71-1492.75) | 585.07 (2559.82) | 513.84 (201.57) | 9.01 (1.31) |
| Neoplasms benign, malignant and unspecified (incl cysts and polyps) | Metastatic carcinoma of the bladder | 4 | 189.46  (69.55-516.08) | 189.33 (717.1) | 181.23  (66.53) | 7.5  (0.97) |
| Neoplasms benign, malignant and unspecified (incl cysts and polyps) | Tumor hyperprogression | 5 | 120.46  (49.5-293.14) | 120.36 (575.39) | 117.04  (48.1) | 6.87 (1.33) |
| Injury, poisoning and procedural complications | Vascular access complication | 8 | 90.47  (44.89-182.34) | 90.35 (692.04) | 88.47  (43.9) | 6.47 (2.07) |
| Infections and infestations | Septic pulmonary embolism | 3 | 85.43  (27.23-268.01) | 85.39 (245.23) | 83.71  (26.68) | 6.39 (0.49) |
| Infections and infestations | Abdominal wall abscess | 4 | 80.68  (29.99-217.05) | 80.62 (308.63) | 79.13  (29.41) | 6.31 (0.94) |
| Investigations | KL-6 increased | 3 | 78.05  (24.9-244.61) | 78.01 (223.92) | 76.61  (24.44) | 6.26 (0.48) |
| Renal and urinary disorders | Immune-mediated nephritis | 5 | 69.12  (28.55-167.31) | 69.06 (329.95) | 67.96  (28.07) | 6.09 (1.29) |
| Neoplasms benign, malignant and unspecified (incl cysts and polyps) | Tumor associated fever | 3 | 61.98  (19.82-193.83) | 61.95 (177.29) | 61.06  (19.53) | 5.93 (0.47) |

**Supplementary Table S5. (Continued)**

| **SOC name** | **PT** | **N** | **ROR (95% CI)** | **PRR (χ^2^)** | **EBGM (EBGM05)** | **IC (IC025)** |
| --- | --- | --- | --- | --- | --- | --- |
| General disorders and administration site conditions | Tissue infiltration | 3 | 61.68  (19.72-192.88) | 61.65 (176.41) | 60.77  (19.43) | 5.93 (0.47) |
| Investigations | Eastern cooperative oncology group performance status worsened | 10 | 54.94  (29.43-102.58) | 54.85 (521.9) | 54.16  (29.01) | 5.76 (2.34) |
| Skin and subcutaneous tissue disorders | SJS–TEN overlap | 6 | 51.95  (23.22-116.26) | 51.9 (295.87) | 51.28  (22.92) | 5.68 (1.55) |
| Skin and subcutaneous tissue disorders | Epidermal necrosis | 4 | 46.58  (17.38-124.82) | 46.55 (176.33) | 46.05  (17.18) | 5.53  (0.9) |
| Neoplasms benign, malignant and unspecified (incl cysts and polyps) | Transitional cell carcinoma | 6 | 45.18  (20.2-101.04) | 45.13 (256.2) | 44.67  (19.97) | 5.48 (1.53) |
| Gastrointestinal disorders | Immune-mediated enterocolitis | 25 | 40.8  (27.49-60.55) | 40.63 (957.23) | 40.25  (27.12) | 5.33 (3.43) |
| Skin and subcutaneous tissue disorders | Dermatitis exfoliative | 5 | 38.75  (16.06-93.51) | 38.72 (182.05) | 38.37  (15.9) | 5.26 (1.22) |
| Immune system disorders | Immune-mediated adverse reaction | 5 | 37.11  (15.38-89.54) | 37.08 (174.02) | 36.77 (15.24) | 5.2 (1.22) |
| Skin and subcutaneous tissue disorders | Skin toxicity | 22 | 37.03  (24.32-56.39) | 36.89 (761.65) | 36.58 (24.02) | 5.19 (3.24) |
| Skin and subcutaneous tissue disorders | Stevens-Johnson syndrome | 49 | 36.77  (27.56-49.05) | 36.48 (1608.06) | 36.17 (27.11) | 5.18 (3.96) |
| Nervous system disorders | Immune-mediated encephalitis | 3 | 36.76  (11.79-114.56) | 36.74 (103.39) | 36.43 (11.69) | 5.19 (0.43) |

**Supplementary Table S5. (Continued)**

| **SOC name** | **PT** | **N** | **ROR (95% CI)** | **PRR (χ^2^)** | **EBGM (EBGM05)** | **IC (IC025)** |
| --- | --- | --- | --- | --- | --- | --- |
| Skin and subcutaneous tissue disorders | Dermatitis bullous | 20 | 35.59  (22.9-55.31) | 35.47 (664.5) | 35.19  (22.64) | 5.14 (3.11) |
| Renal and urinary disorders | Urinary tract obstruction | 7 | 32.95  (15.66-69.35) | 32.91 (214.91) | 32.66  (15.52) | 5.03 (1.69) |
| Skin and subcutaneous tissue disorders | Symmetrical drug-related intertriginous and flexural exanthema | 4 | 28.39  (10.61-75.91) | 28.37 (104.9) | 28.18  (10.54) | 4.82 (0.83) |
| Hepatobiliary disorders | Immune-mediated hepatic disorder | 10 | 27.85  (14.95-51.9) | 27.81 (256.73) | 27.63  (14.83) | 4.79 (2.14) |
| Respiratory, thoracic and mediastinal disorders | Immune-mediated lung disease | 9 | 26.57  (13.79-51.2) | 26.53 (219.74) | 26.37  (13.69) | 4.72 (1.98) |
| Respiratory, thoracic and mediastinal disorders | Pulmonary toxicity | 20 | 25.25  (16.26-39.22) | 25.16 (461.39) | 25.02  (16.11) | 4.65 (2.91) |
| Nervous system disorders | Chronic inflammatory demyelinating polyradiculoneuropathy | 4 | 24.8  (9.28-66.28) | 24.78 (90.75) | 24.64  (9.22) | 4.62 (0.81) |
| Skin and subcutaneous tissue disorders | Toxic epidermal necrolysis | 30 | 24.29  (16.95-34.81) | 24.17 (662.76) | 24.04  (16.78) | 4.59 (3.26) |
| General disorders and administration site conditions | Administration site extravasation | 3 | 23.28  (7.48-72.44) | 23.27  (63.6) | 23.15  (7.44) | 4.53 (0.38) |

**Supplementary Table S6. Top 30 PT-level ADEs sorted by IC for EV + Pembro in VigiBase**

| **SOC name** | **PT** | **N** | **ROR (95% CI)** | **PRR (χ^2^)** | **EBGM (EBGM05)** | **IC (IC025)** |
| --- | --- | --- | --- | --- | --- | --- |
| Skin and subcutaneous tissue disorders | Toxic erythema of chemotherapy | 3 | 461.08  (146.73-1448.9) | 459.54 (1345.19) | 450.37 (172.78) | 8.81 (7.13) |
| Skin and subcutaneous tissue disorders | Epidermal necrosis | 4 | 351.64  (130.69-946.14) | 350.08 (1371.0) | 344.73 (150.59) | 8.43 (6.75) |
| Skin and subcutaneous tissue disorders | Skin toxicity | 33 | 291.92  (205.8-414.1) | 281.54 (910.59) | 277.97 (207.47) | 8.12 (6.45) |
| Skin and subcutaneous tissue disorders | Symmetrical drug-related intertriginous and flexural exanthema | 5 | 186.1  (76.99-449.84) | 185.07 (907.94) | 183.57 (87.72) | 7.52 (5.85) |
| Skin and subcutaneous tissue disorders | SJS–TEN overlap | 4 | 173.79  (64.84-465.83) | 173.02 (678.91) | 171.71 (75.25) | 7.42 (5.75) |
| Immune system disorders | Immune-mediated adverse reaction | 3 | 124.14  (39.84-386.84) | 123.73 (363.21) | 123.05 (47.54) | 6.94 (5.27) |
| Nervous system disorders | Peripheral motor neuropathy | 3 | 122.79  (39.4-382.62) | 122.38 (359.22) | 121.72 (47.03) | 6.93 (5.25) |
| Metabolism and nutrition disorders | Insulin resistance | 5 | 103.64  (42.94-250.11) | 103.07 (503.1) | 102.6  (49.09) | 6.68 (5.01) |
| Skin and subcutaneous tissue disorders | Dermatitis bullous | 22 | 88.33  (57.82-134.95) | 86.23 (1846.63) | 85.9  (60.25) | 6.42 (4.76) |
| Surgical and medical procedures | Radiotherapy | 4 | 78.93  (29.51-211.12) | 78.58 (305.32) | 78.31  (34.38) | 6.29 (4.62) |
| Nervous system disorders | Peripheral sensory neuropathy | 8 | 64.34  (32.05-129.19) | 63.78 (493.05) | 63.6  (35.5) | 5.99 (4.32) |
| Hepatobiliary disorders | Immune-mediated hepatitis | 4 | 63.96  (23.92-171.02) | 63.68 (246.09) | 63.5  (27.88) | 5.99 (4.32) |
| Skin and subcutaneous tissue disorders | Toxic epidermal necrolysis | 15 | 62.26  (37.35-103.79) | 61.25 (886.79) | 61.08  (39.83) | 5.93 (4.26) |
| Respiratory, thoracic and mediastinal disorders | Immune-mediated lung disease | 4 | 56.1  (20.98-149.98) | 55.85 (214.96) | 55.72  (24.47) | 5.8  (4.13) |

**Supplementary Table S6. (Continued)**

| **SOC name** | **PT** | **N** | **ROR (95% CI)** | **PRR (χ^2^)** | **EBGM (EBGM05)** | **IC (IC025)** |
| --- | --- | --- | --- | --- | --- | --- |
| Skin and subcutaneous tissue disorders | Stevens-Johnson syndrome | 29 | 54.61  (37.71-79.08) | 52.92 (1474.58) | 52.8  (38.73) | 5.72 (4.05) |
| General disorders and administration site conditions | Therapy partial responder | 12 | 45.42  (25.68-80.33) | 44.83 (513.36) | 44.74  (27.77) | 5.48 (3.81) |
| Nervous system disorders | Neuropathy peripheral | 112 | 48.83  (40.11-59.44) | 43.49 (4651.71) | 43.4 (36.82) | 5.44 (3.77) |
| Skin and subcutaneous tissue disorders | Dermatitis exfoliative | 4 | 43.34 (16.22-115.85) | 43.16 (164.41) | 43.07 (18.92) | 5.43 (3.76) |
| Nervous system disorders | Polyneuropathy | 13 | 42.22  (24.41-73.04) | 41.63 (514.74) | 41.56 (26.27) | 5.38 (3.71) |
| Respiratory, thoracic and mediastinal disorders | Pneumonitis | 26 | 42.52  (28.78-62.82) | 41.34 (1022.29) | 41.27 (29.77) | 5.37 (3.7) |
| Neoplasms benign, malignant and unspecified (incl cysts and polyps) | Malignant neoplasm progression | 72 | 41.18  (32.38-52.37) | 38.18 (2607.02) | 38.11 (31.17) | 5.25 (3.58) |
| Respiratory, thoracic and mediastinal disorders | Organising pneumonia | 3 | 37.28  (11.99-115.93) | 37.16 (105.4) | 37.1 (14.36) | 5.21 (3.54) |
| Metabolism and nutrition disorders | Diabetic ketoacidosis | 12 | 34.11  (19.29-60.32) | 33.67 (380.0) | 33.62 (20.87) | 5.07 (3.4) |
| Infections and infestations | Urosepsis | 3 | 29.66  (9.54-92.22) | 29.57 (82.7) | 29.53 (11.43) | 4.88 (3.21) |
| General disorders and administration site conditions | Infusion site extravasation | 5 | 29.12  (12.08-70.18) | 28.97 (134.85) | 28.93 (13.86) | 4.85 (3.19) |
| Hepatobiliary disorders | Hepatitis | 15 | 28.99  (17.4-48.31) | 28.53 (398.19) | 28.49 (18.59) | 4.83 (3.16) |
| Respiratory, thoracic and mediastinal disorders | Lung opacity | 5 | 28.33  (11.75-68.26) | 28.17 (130.91) | 28.14 (13.48) | 4.81 (3.15) |
| Nervous system disorders | Myasthenia gravis | 5 | 27.8  (11.54-67.0) | 27.65 (128.32) | 27.62 (13.23) | 4.79 (3.12) |

**Supplementary Table S6. (Continued)**

| **SOC name** | **PT** | **N** | **ROR (95% CI)** | **PRR (χ^2^)** | **EBGM (EBGM05)** | **IC (IC025)** |
| --- | --- | --- | --- | --- | --- | --- |
| Metabolism and nutrition disorders | Hypophosphatemia | 4 | 27.33  (10.23-73.02) | 27.21 (100.88) | 27.18  (11.94) | 4.76 (3.09) |
| General disorders and administration site conditions | Multiple organ dysfunction syndrome | 9 | 24.56  (12.73-47.38) | 24.33 (201.19) | 24.3  (14.03) | 4.6  (2.93) |

**Supplementary Table S7. Top 30 PT-level ADEs sorted by IC for EV + Pembro in FAERS**

| **SOC name** | **PT** | **N** | **ROR (95% CI)** | **PRR (χ^2^)** | **EBGM (EBGM05)** | **IC (IC025)** |
| --- | --- | --- | --- | --- | --- | --- |
| Infections and infestations | Pleurisy bacterial | 3 | 632.2  (187.81-2128.11) | 631.87 (1643.15) | 549.58 (163.27) | 9.1  (0.43) |
| Neoplasms benign, malignant and unspecified (incl cysts and polyps) | Transitional cell carcinoma metastatic | 5 | 585.57  (229.71-1492.75) | 585.07 (2559.82) | 513.84 (201.57) | 9.01 (1.31) |
| Neoplasms benign, malignant and unspecified (incl cysts and polyps) | Metastatic carcinoma of the bladder | 4 | 189.46  (69.55-516.08) | 189.33 (717.1) | 181.23 (66.53) | 7.5  (0.97) |
| Neoplasms benign, malignant and unspecified (incl cysts and polyps) | Tumor hyperprogression | 5 | 120.46  (49.5-293.14) | 120.36 (575.39) | 117.04  (48.1) | 6.87 (1.33) |
| Injury, poisoning and procedural complications | Vascular access complication | 8 | 90.47  (44.89-182.34) | 90.35 (692.04) | 88.47  (43.9) | 6.47 (2.07) |
| Infections and infestations | Septic pulmonary embolism | 3 | 85.43  (27.23-268.01) | 85.39 (245.23) | 83.71  (26.68) | 6.39 (0.49) |
| Infections and infestations | Abdominal wall abscess | 4 | 80.68  (29.99-217.05) | 80.62 (308.63) | 79.13  (29.41) | 6.31 (0.94) |
| Investigations | KL-6 increased | 3 | 78.05  (24.9-244.61) | 78.01 (223.92) | 76.61  (24.44) | 6.26 (0.48) |
| Renal and urinary disorders | Immune-mediated nephritis | 5 | 69.12  (28.55-167.31) | 69.06 (329.95) | 67.96  (28.07) | 6.09 (1.29) |
| Neoplasms benign, malignant and unspecified (incl cysts and polyps) | Tumor associated fever | 3 | 61.98  (19.82-193.83) | 61.95 (177.29) | 61.06  (19.53) | 5.93 (0.47) |
| General disorders and administration site conditions | Tissue infiltration | 3 | 61.68  (19.72-192.88) | 61.65 (176.41) | 60.77  (19.43) | 5.93 (0.47) |
| Investigations | Eastern cooperative oncology group performance status worsened | 10 | 54.94  (29.43-102.58) | 54.85 (521.9) | 54.16  (29.01) | 5.76 (2.34) |

**Supplementary Table S7. (Continued)**

| **SOC name** | **PT** | **N** | **ROR (95% CI)** | **PRR (χ^2^)** | **EBGM (EBGM05)** | **IC (IC025)** |
| --- | --- | --- | --- | --- | --- | --- |
| Skin and subcutaneous tissue disorders | SJS–TEN overlap | 6 | 51.95  (23.22-116.26) | 51.9 (295.87) | 51.28  (22.92) | 5.68 (1.55) |
| Skin and subcutaneous tissue disorders | Epidermal necrosis | 4 | 46.58  (17.38-124.82) | 46.55 (176.33) | 46.05  (17.18) | 5.53  (0.9) |
| Neoplasms benign, malignant and unspecified (incl cysts and polyps) | Transitional cell carcinoma | 6 | 45.18  (20.2-101.04) | 45.13 (256.2) | 44.67  (19.97) | 5.48 (1.53) |
| Gastrointestinal disorders | Immune-mediated enterocolitis | 25 | 40.8  (27.49-60.55) | 40.63 (957.23) | 40.25 (27.12) | 5.33 (3.43) |
| Skin and subcutaneous tissue disorders | Dermatitis exfoliative | 5 | 38.75  (16.06-93.51) | 38.72 (182.05) | 38.37 (15.9) | 5.26 (1.22) |
| Immune system disorders | Immune-mediated adverse reaction | 5 | 37.11  (15.38-89.54) | 37.08 (174.02) | 36.77 (15.24) | 5.2 (1.22) |
| Skin and subcutaneous tissue disorders | Skin toxicity | 22 | 37.03  (24.32-56.39) | 36.89 (761.65) | 36.58 (24.02) | 5.19 (3.24) |
| Nervous system disorders | Immune-mediated encephalitis | 3 | 36.76  (11.79-114.56) | 36.74 (103.39) | 36.43 (11.69) | 5.19 (0.43) |
| Skin and subcutaneous tissue disorders | Stevens-Johnson syndrome | 49 | 36.77  (27.56-49.05) | 36.48 (1608.06) | 36.17 (27.11) | 5.18 (3.96) |
| Skin and subcutaneous tissue disorders | Dermatitis bullous | 20 | 35.59  (22.9-55.31) | 35.47 (664.5) | 35.19 (22.64) | 5.14 (3.11) |
| Renal and urinary disorders | Urinary tract obstruction | 7 | 32.95  (15.66-69.35) | 32.91 (214.91) | 32.66 (15.52) | 5.03 (1.69) |
| Skin and subcutaneous tissue disorders | Symmetrical drug-related intertriginous and flexural exanthema | 4 | 28.39  (10.61-75.91) | 28.37 (104.9) | 28.18 (10.54) | 4.82 (0.83) |

**Supplementary Table S7. (Continued)**

| **SOC name** | **PT** | **N** | **ROR (95% CI)** | **PRR (χ^2^)** | **EBGM (EBGM05)** | **IC (IC025)** |
| --- | --- | --- | --- | --- | --- | --- |
| Hepatobiliary disorders | Immune-mediated hepatic disorder | 10 | 27.85  (14.95-51.9) | 27.81 (256.73) | 27.63 (14.83) | 4.79 (2.14) |
| Respiratory, thoracic and mediastinal disorders | Immune-mediated lung disease | 9 | 26.57  (13.79-51.2) | 26.53 (219.74) | 26.37 (13.69) | 4.72 (1.98) |
| Respiratory, thoracic and mediastinal disorders | Pulmonary toxicity | 20 | 25.25  (16.26-39.22) | 25.16 (461.39) | 25.02 (16.11) | 4.65 (2.91) |
| Nervous system disorders | Chronic inflammatory demyelinating polyradiculoneuropathy | 4 | 24.8  (9.28-66.28) | 24.78 (90.75) | 24.64 (9.22) | 4.62 (0.81) |
| Skin and subcutaneous tissue disorders | Toxic epidermal necrolysis | 30 | 24.29  (16.95-34.81) | 24.17 (662.76) | 24.04 (16.78) | 4.59 (3.26) |
| General disorders and administration site conditions | Administration site extravasation | 3 | 23.28  (7.48-72.44) | 23.27 (63.6) | 23.15 (7.44) | 4.53 (0.38) |

**Supplementary Table S8. Sex-exclusive PTs by database (male-only vs female-only)**

| **PTs were reported only**  **in female** | **VigiBase** | **FAERS** | **PTs were reported only in male** | **VigiBase** | **FAERS** |
| --- | --- | --- | --- | --- | --- |
| Anemia of malignant disease | 2 | 1 | Blood creatinine increased | 6 | 13 |
| Blindness unilateral | 2 | 3 | Septic shock | 6 | 6 |
| Organ failure | 1 | 2 | Transaminases increased | 5 | 6 |
| Epistaxis | 1 | 2 | Ill-defined disorder | 4 | 7 |
| Skin ulcer | 1 | 2 | Colitis ulcerative | 5 | 4 |
| Presyncope | 1 | 2 | Symmetrical drug-related intertriginous and flexural exanthema | 5 | 4 |
| Anaphylactic shock | 1 | 2 | Somnolence | 3 | 6 |
| Cardiac arrest | 1 | 2 | Leukopenia | 2 | 7 |
| Hyperlipidaemia | 1 | 2 | Blood urine present | 2 | 6 |
| Increased bronchial secretion | 1 | 2 | Cardiac disorder | 3 | 5 |
| Leukaemia | 1 | 2 | Epidermal necrosis | 4 | 4 |
| Vaginal haemorrhage | 1 | 2 | Tubulointerstitial nephritis | 4 | 4 |

**Supplementary Table S8. (Continued)**

| **PTs were reported only**  **in female** | **VigiBase** | **FAERS** | **PTs were reported only in male** | **VigiBase** | **FAERS** |
| --- | --- | --- | --- | --- | --- |
| Ventricular fibrillation | 1 | 2 | Gait disturbance | 3 | 5 |
|  |  |  | Muscular weakness | 3 | 5 |
|  |  |  | Feeding disorder | 2 | 6 |
|  |  |  | Hemoglobin decreased | 2 | 6 |

**Supplementary Table S9. Analysis of sex-differentiated risk signals in EV + Pembro**

| **Database** | **SOC** | **PT** | **Female/Male** | **ROR(95% CI)** | **P value** |
| --- | --- | --- | --- | --- | --- |
| VigiBase | Skin and subcutaneous tissue disorders | Skin discoloration | 7/1 | 22.18 (2.71-181.31) | 0.00032 |
| VigiBase | Skin and subcutaneous tissue disorders | Alopecia | 16/17 | 2.98 (1.48-6.01) | 0.00140 |
| VigiBase | Skin and subcutaneous tissue disorders | Stevens-Johnson syndrome | 14/15 | 2.96 (1.40-6.23) | 0.00288 |
| VigiBase | Investigations | Hepatic enzyme increased | 6/4 | 4.75 (1.33-17.00) | 0.01693 |
| VigiBase | Gastrointestinal disorders | Nausea | 10/11 | 2.88 (1.21-6.88) | 0.01292 |
| VigiBase | General disorders and administration site conditions | Fatigue | 17/26 | 2.07 (1.10-3.89) | 0.02110 |
| VigiBase | Skin and subcutaneous tissue disorders | Rash | 36/70 | 1.63 (1.06-2.51) | 0.02537 |
| FAERS | Gastrointestinal disorders | Gastroesophageal reflux disease | 6/1 | 17.06 (2.05-141.86) | 0.00170 |
| FAERS | Skin and subcutaneous tissue disorders | Alopecia | 35/38 | 2.65 (1.67-4.21) | 0.00002 |
| FAERS | Respiratory, thoracic and mediastinal disorders | Pulmonary oedema | 5/1 | 14.21 (1.66-121.73) | 0.00565 |
| FAERS | Skin and subcutaneous tissue disorders | Skin discoloration | 7/4 | 4.98 (1.45-17.03) | 0.00965 |
| FAERS | Investigations | Hepatic enzyme increased | 11/9 | 3.47 (1.43-8.39) | 0.00328 |

**Supplementary Table S9. (Continued)**

| **Database** | **SOC** | **PT** | **Female/Male** | **ROR(95% CI)** | **P value** |
| --- | --- | --- | --- | --- | --- |
| FAERS | General disorders and administration site conditions | Therapy non-responder | 5/2 | 7.10 (1.38-36.65) | 0.01552 |
| FAERS | Investigations | Blood magnesium decreased | 4/1 | 11.36 (1.27-101.72) | 0.01828 |
| FAERS | Musculoskeletal and connective tissue disorders | Joint swelling | 4/1 | 11.36 (1.27-101.72) | 0.01828 |
| FAERS | Skin and subcutaneous tissue disorders | Eczema | 7/5 | 3.98 (1.26-12.56) | 0.01799 |
| FAERS | Eye disorders | Dry eye | 11/11 | 2.85 (1.23-6.58) | 0.01050 |
| FAERS | Investigations | Oxygen saturation decreased | 6/4 | 4.26 (1.20-15.13) | 0.02421 |
| FAERS | Musculoskeletal and connective tissue disorders | Pain in extremity | 7/6 | 3.32 (1.11-9.88) | 0.04988 |
| FAERS | Skin and subcutaneous tissue disorders | Stevens-Johnson syndrome | 19/30 | 1.80 (1.01-3.21) | 0.04272 |
| FAERS | Skin and subcutaneous tissue disorders | Pruritus | 40/75 | 1.53 (1.03-2.25) | 0.03196 |
| FAERS | Metabolism and nutrition disorders | Dehydration | 1/30 | 0.09 (0.01-0.69) | 0.00365 |
| FAERS | Renal and urinary disorders | Renal impairment | 2/26 | 0.22 (0.05-0.91) | 0.02209 |

**Supplementary Table S10. Time-to-onset by SOC in FAERS (median and IQR)**

| **SOC** | **Median (Q1–Q3)** |
| --- | --- |
| Immune system disorders | 9 (9-33.75) |
| Musculoskeletal and connective tissue disorders | 9.5 (7-15.5) |
| Investigations | 10 (6.25-20.75) |
| Eye disorders | 10 (7-21.25) |
| Gastrointestinal disorders | 10 (7-32.25) |
| Blood and lymphatic system disorders | 10 (7-21) |
| Skin and subcutaneous tissue disorders | 10 (7-19) |
| General disorders and administration site conditions | 12 (7-32.5) |
| Renal and urinary disorders | 14 (7-49.75) |
| Metabolism and nutrition disorders | 14 (7-30.5) |
| Injury, poisoning and procedural complications | 14 (6.5-31) |
| Infections and infestations | 14 (6-47) |
| Hepatobiliary disorders | 14 (9-39) |
| Vascular disorders | 14.5 (7-19) |
| Psychiatric disorders | 16 (9-59) |
| Neoplasms benign, malignant and unspecified (incl cysts and polyps) | 17 (7-41.5) |
| Cardiac disorders | 17.5 (7-34) |
| Nervous system disorders | 18 (7-43) |
| Respiratory, thoracic and mediastinal disorders | 21 (8.75-65.5) |
| Endocrine disorders | 28 (14.25-57.5) |
| Surgical and medical procedures | 40.5 (17.75-64) |
